# Shape-shifting thermoreversible diblock copolymer nano-objects *via* RAFT aqueous dispersion polymerization of 4-hydroxybutyl acrylate†

**DOI:** 10.1039/d1sc05022b

**Published:** 2021-10-07

**Authors:** Oliver J. Deane, James Jennings, Steven P. Armes

**Affiliations:** Dainton Building, Department of Chemistry, University of Sheffield Brook Hill Sheffield South Yorkshire S3 7HF UK s.p.armes@sheffield.ac.uk

## Abstract

2-Hydroxypropyl methacrylate (HPMA) is a useful model monomer for understanding aqueous dispersion polymerization. 4-Hydroxybutyl acrylate (HBA) is an isomer of HPMA: it has appreciably higher aqueous solubility so its homopolymer is more weakly hydrophobic. Moreover, PHBA possesses a significantly lower glass transition temperature than PHPMA, which ensures greater chain mobility. The reversible addition–fragmentation chain transfer (RAFT) aqueous dispersion polymerization of HBA using a poly(ethylene glycol) (PEG_113_) precursor at 30 °C produces PEG_113_–PHBA_200–700_ diblock copolymer nano-objects. Using glutaraldehyde to crosslink the PHBA chains allows TEM studies, which reveal the formation of spheres, worms or vesicles under appropriate conditions. Interestingly, the partially hydrated highly mobile PHBA block enabled linear PEG_113_–PHBA_*x*_ spheres, worms or vesicles to be reconstituted from freeze-dried powders on addition of water at 20 °C. Moreover, variable temperature ^1^H NMR studies indicated that the apparent degree of hydration of the PHBA block increases from 5% to 80% on heating from 0 °C to 60 °C indicating uniform plasticization. In contrast, the PHPMA_*x*_ chains within PEG_113_–PHPMA_*x*_ nano-objects become dehydrated on raising the temperature: this qualitative difference is highly counter-intuitive given that PHBA and PHPMA are isomers. The greater (partial) hydration of the PHBA block at higher temperature drives the morphological evolution of PEG_113_–PHBA_260_ spheres to form worms or vesicles, as judged by oscillatory rheology, dynamic light scattering, small-angle X-ray scattering and TEM studies. Finally, a variable temperature phase diagram is constructed for 15% w/w aqueous dispersions of eight PEG_113_–PHBA_200–700_ diblock copolymers. Notably, PEG_113_–PHBA_350_ can switch reversibly from spheres to worms to vesicles to lamellae during a thermal cycle.

## Abstract 2

RAFT aqueous dispersion polymerization of 4-hydroxybutyl acrylate (HBA) affords shape-shifting thermoresponsive diblock copolymer nano-objects. ^1^H NMR studies suggest that such behavior involves uniform plasticization of the PHBA block.

## Graphical abstract

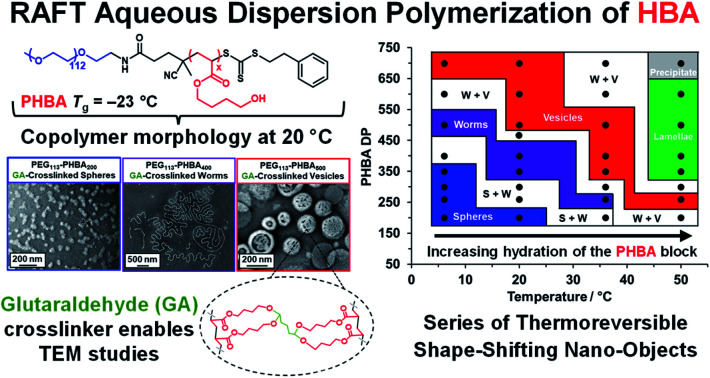

## Introduction

**Fig. 1:**
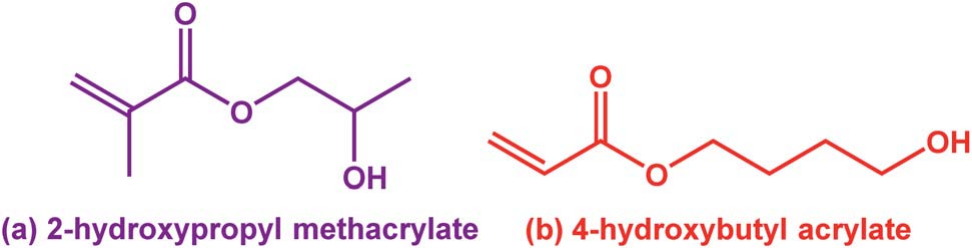
Chemical structures for (a) 2-hydroxypropyl methacrylate (HPMA; only the major isomer is shown in this case) and (b) its acrylic isomer, 4-hydroxybutyl acrylate (HBA).

Diblock copolymer self-assembly in a solvent that is selective for one of the two blocks enables the preparation of sterically-stabilized nano-objects that have been utilized for a broad range of applications.^1–8^ Traditionally, living anionic polymerization has been used to prepare molecularly-dissolved copolymer chains prior to their isolation, purification and self-assembly *via* various post-polymerization techniques, typically in dilute solution.^1–3,5,6,9^ Over the past two decades or so, polymerization-induced self-assembly (PISA) has become widely recognized as a powerful platform technology for the rational synthesis of many block copolymer nano-objects.^10–16^ PISA is both efficient and versatile: in essence, it involves growing an insoluble second block from a soluble precursor block in a suitable selective solvent. PISA can be performed in many solvents, including water. Most aqueous PISA formulations involve RAFT polymerization, which is highly tolerant of both protic solvents and many types of functional vinyl monomers.^17–20^ Depending on whether the monomer chosen for the second block is water-immiscible or water-miscible, either RAFT emulsion^21–26^ or RAFT dispersion^27–39^ polymerization can be employed. However, typically only the latter formulation provides access to thermoresponsive diblock copolymer worms and vesicles.^7,40–50^ Variable temperature ^1^H NMR studies have confirmed that such stimulus-responsive behavior involves a subtle change in the partial degree of hydration of the core-forming block.^50,51^ Various aqueous thermoresponsive diblock copolymer formulations involve using 2-hydroxypropyl methacrylate (HPMA; Fig. 1a) to produce a weakly hydrophobic core-forming PHPMA block.^28,32,52–54^

Essentially, the HPMA repeat units nearest the block junction become hydrated, which leads to a shift in the effective block junction. This is sufficient to lower the fractional packing parameter *P* from the relatively narrow range that favors worms (0.33 < *P* ≤ 0.50) to that corresponding to spheres (*P* ≤ 0.33). Importantly, this morphological transition proved to be reversible, which has enabled various cell biology studies to be explored for worm gels.^7,32,55,56^ Similarly, macromolecular RAFT agents based on monomethoxy-capped poly(ethylene glycol) (PEG) have been used by various research groups to design thermoresponsive PEG–PHPMA nano-objects.^52,53,57,58^ More specifically, esterification enables the synthesis of well-defined PEG precursors with minimal batch-to-batch variation.^52,53,56–58^ Furthermore, using PEG as a steric stabilizer ensures well-resolved proton signals for each block, which in turn should facilitate variable temperature ^1^H NMR spectroscopy studies.

In principle, an alternative route to thermoreversible PEG-based diblock copolymer nano-objects could be achieved by employing a less hydrophobic vinyl monomer that exhibits greater thermoresponsive behavior.^50,59^ Recently, Byard *et al.* reported the one-pot PISA synthesis of poly(*N*,*N*-dimethylacrylamide)–poly(4-hydroxybutyl acrylate-*stat*-diacetone acrylamide) [PDMAC_56_–P(HBA-*stat*-DAAM)_264_].^50^ This amphiphilic diblock copolymer exhibited remarkable self-assembly behavior, with thermoreversible transitions between spheres, worms, vesicles and lamellae being observed in aqueous solution for a single copolymer simply by adjusting the temperature.^50^ Variable temperature ^1^H NMR studies indicated that the weakly hydrophobic HBA repeat units became more hydrated at elevated temperatures. This seems rather counter-intuitive: precisely the opposite behavior is observed for PHPMA-based diblock copolymers, for which the HPMA repeat units become marginally less hydrated on heating.^7,50^ This apparent difference is particularly striking given that HPMA and HBA are structural isomers (Fig. 1). Unfortunately, the statistical copolymerization of 20 mol% DAAM comonomer with HBA by Byard *et al.* prevents a direct comparison of the thermoresponsive behavior of PHBA and PHPMA. This problem is resolved in the present study.

**Scheme 1:**
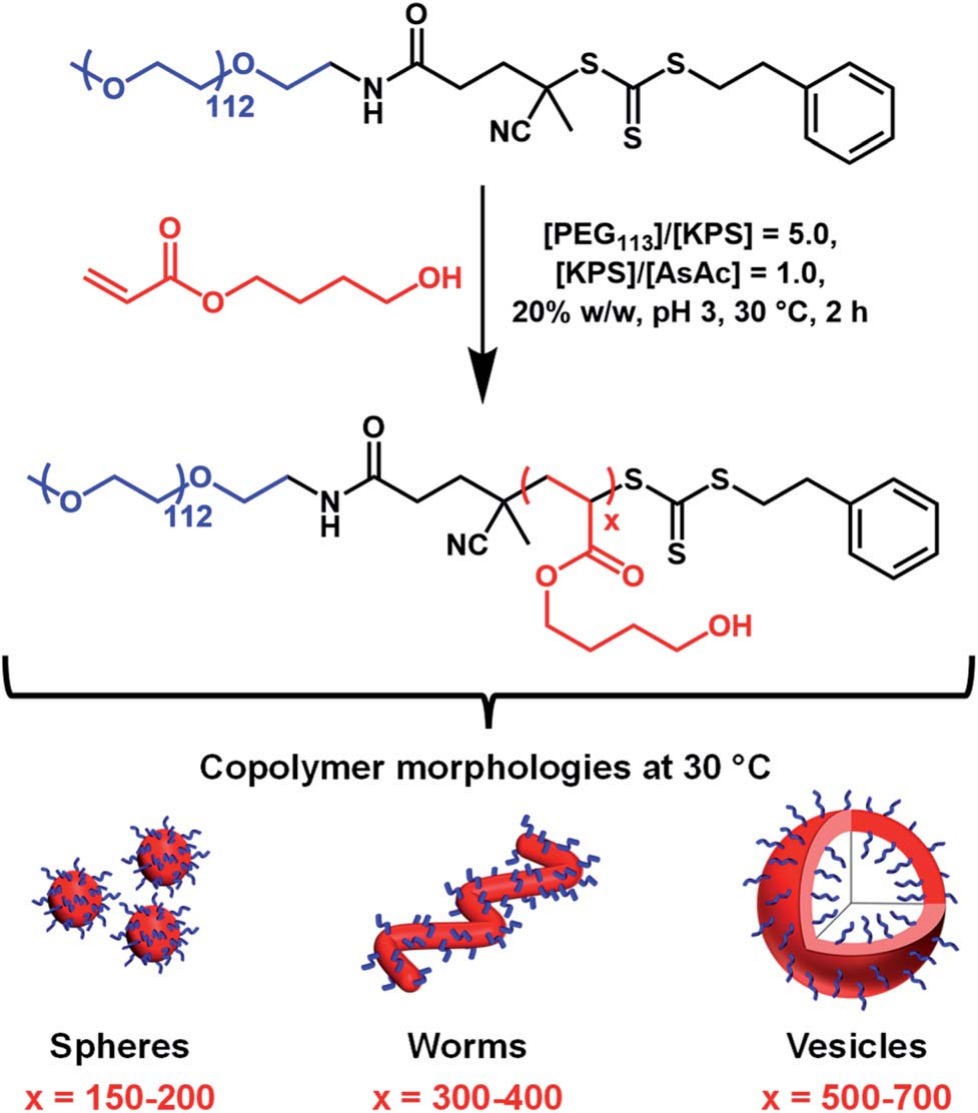
Synthesis of PEG_113_–PHBA_*x*_ nano-objects *via* RAFT dispersion polymerization of HBA at 30 °C using a potassium persulfate (KPS) plus ascorbic acid (AsAc) redox initiator pair ([KPS]/[AsAc] molar ratio = 1.0). The [HBA]/[PEG_113_] molar ratio was varied between 200 and 700 while the [PEG_113_]/[KPS] molar ratio was fixed at 5.0 for all polymerizations. The three schematic cartoons of spheres, worms and vesicles refer to copolymer morphologies observed at 30 °C.

Herein we report the RAFT aqueous dispersion polymerization of HBA using a PEG_113_ precursor to afford thermoresponsive diblock copolymer spheres, worms or vesicles at 30 °C (Scheme 1). TEM studies of such low *T*_g_ nano-objects requires chemical crosslinking of the PHBA chains *via* their pendent hydroxyl groups.^60^ Such covalent stabilization enables the construction of a pseudo-phase diagram using TEM to assign copolymer morphologies. The thermoreversible behavior of linear PEG_113_–PHBA_260_ nano-objects is assessed using oscillatory rheology, dynamic light scattering (DLS) and small-angle X-ray scattering (SAXS) and is explicitly compared to that of PEG_113_–PHPMA_260_ nano-objects. Variable temperature ^1^H NMR spectroscopy is used to monitor the degree of hydration of the PHBA block and the reconstitution of freeze-dried PEG_113_–PHBA_*x*_ powders to form aqueous dispersions of diblock copolymer nano-objects at neutral pH is examined at 20 °C. Finally, a first-of-its-kind variable temperature phase diagram is constructed to highlight the copolymer morphologies that are accessible for such PEG_113_–PHBA_*x*_ diblock copolymers.

## Results and discussion

The PEG_113_–PHBA_*x*_ diblock copolymers used in this study were synthesized *via* RAFT aqueous dispersion polymerization of HBA using a previously reported trithiocarbonate-based PEG_113_ precursor^53^ (Scheme 1). ^1^H NMR studies (CD_3_OD) of the molecularly-dissolved PEG_113_-PHBA_*x*_ diblock copolymers confirmed that high HBA conversions (>99%) were routinely achieved within 2 h at 30 °C. DMF GPC analysis of three PEG_113_–PHBA_*x*_ diblock copolymers (where *x* = 200, 400 and 700; target solids concentration = 20% w/w) indicated high blocking efficiencies and relatively narrow molecular weight distributions were achieved (*M*_w_/*M*_n_ = 1.21, 1.24 or 1.45; Fig. S1a†). Increasing the DP of the PHBA block had a significant effect on the visual appearance of 20% w/w aqueous dispersions of PEG_113_–PHBA_200–700_ nano-objects. For example, PEG_113_–PHBA_200_ was a transparent free-flowing fluid at 20 °C, whereas PEG_113_–PHBA_400_ formed a free-standing gel and the PEG_113_–PHBA_700_ dispersion was free-flowing but highly turbid. Comparing these observations with those reported by Warren *et al.* for PEG_113_–PHPMA_*x*_ dispersions suggested the successful synthesis of spheres, worms and vesicles, respectively. However, the PHBA DP required to afford each morphology was significantly higher than that required for PHPMA-based nano-objects.^52^

To further examine the difference between these two PISA formulations, 15% w/w aqueous dispersions of PEG_113_–PHPMA_260_ and PEG_113_–PHBA_260_ nano-objects were prepared *via* RAFT aqueous dispersion polymerization at 30 °C. ^1^H NMR analysis confirmed that a DP of 260 was achieved in each case. DMF GPC analysis indicated similar *M*_n_ values and dispersities for PEG_113_–PHBA_260_ and PEG_113_–PHPMA_260_ (Fig. S1b†). Given the high blocking efficiencies and essentially full monomer conversions, the small difference in *M*_n_ values is attributed to a slightly larger hydrodynamic radius for PEG_113_–PHPMA_260_ chains in DMF. Visual inspection indicated that the PEG_113_–PHBA_260_ nano-objects formed a free-flowing, highly transparent fluid at 20 °C, whereas the PEG_113_–PHPMA_260_ nano-objects formed a viscous turbid fluid at the same temperature.

**Fig. 2:**
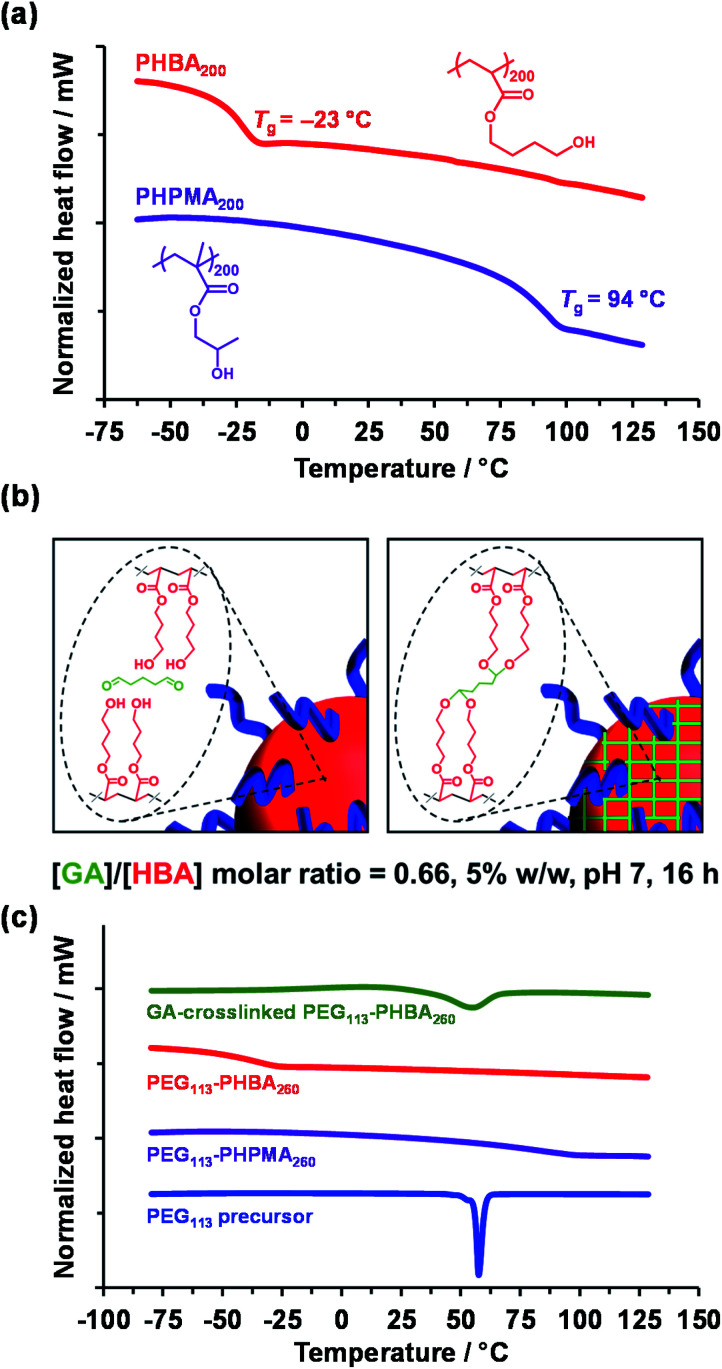
(a) DSC curves recorded at 10 °C min^−1^ for PHBA_200_ (red trace) and PHPMA_200_ (purple trace) homopolymers prepared *via* RAFT solution polymerization in methanol. (b) Schematic cartoon illustrating the intermolecular crosslinking of the PHBA chains within PEG_113_–PHBA_*x*_ nano-objects *via* attack of the pendent hydroxyl groups using excess glutaraldehyde (GA). (c) DSC curves recorded at 10 °C min^−1^ for GA-crosslinked PEG_113_–PHBA_260_ (green trace; [GA]/[HBA] molar ratio = 0.66 at 20 °C for 16 h), linear PEG_113_–PHBA_260_ (red trace), linear PEG_113_–PHBA_260_ (purple trace) and the PEG_113_ precursor (blue trace). Each (co)polymer was freeze-dried for 24 h and subsequently dried at 30 °C under vacuum for three days prior to analysis. DSC curves are arbitrarily offset for clarity.

TEM analysis of PHPMA-based nano-objects is well established in the literature.^28,34,52,61–65^ Differential scanning calorimetry (DSC) studies indicated a relatively high *T*_g_ of 94 °C after drying under vacuum for three days at 30 °C (Fig. 2a, purple trace) for a PHPMA_200_ homopolymer, which is consistent with the literature.^28^ This *T*_g_ enabled high-quality TEM images to be readily obtained when using a heavy metal stain to enhance electron contrast. In contrast, the sub-ambient *T*_g_ of −23 °C indicated by DSC analysis of PHBA_200_ homopolymer (Fig. 2a, red trace) prevents TEM analysis of linear PEG_113_–PHBA_260_ nano-objects owing to nanoparticle deformation and/or film formation during grid preparation. Moreover, the partially hydrated nature of the PHBA chains leads to poor electron contrast when using cryo-TEM.^39^ Further DSC studies indicated that the *T*_g_ of PHBA has a rather weak molecular weight dependence (Fig. S2†).

Glutaraldehyde (GA) is a well-known fixative that is commonly used to crosslink biological samples such as proteins prior to TEM studies.^66–69^ The aqueous chemistry of GA is rather complex: depending on the solution pH, a mixture of monomeric GA, polymeric GA and cyclic GA species can be formed.^70^ Nevertheless, the two aldehyde groups on GA can react efficiently with adjacent hydroxy groups on poly(vinyl alcohol) (PVA).^71,72^ Very recently, we found that GA provides sufficient covalent stabilization of PHBA-based nano-objects to enable good-quality TEM images to be obtained.^60^ Moreover, by avoiding copolymerization of HBA with crosslinkable comonomers such as DAAM, this approach facilitates direct comparison of the thermoresponsive behavior of PHPMA-based and PHBA-based nano-objects.

In the present study, the [GA]/[HBA] molar ratio was systematically varied from 0.25 to 1.00 when crosslinking a 5% w/w aqueous dispersion of PEG_113_–PHBA_260_ nano-objects. Empirically, the optimum [GA]/[HBA] molar ratio was found to be 0.66, which is significantly higher than the stoichiometric ratio of 0.25 (Fig. 2b). Moreover, using more concentrated (>7.5% w/w) aqueous dispersions led to irreversible macroscopic gelation owing to inter-particle crosslinking. DSC and FT-IR spectroscopy studies were undertaken to examine the effect of GA crosslinking on the PEG_113_–PHBA_*x*_ diblock copolymers. Initially, linear PEG_113_–PHBA_260_ and PEG_113_–PHPMA_260_ were freeze-dried to afford a tacky gum and a glassy powder, respectively. DSC studies indicated *T*_g_ values of 85 °C for PEG_113_–PHPMA_260_ and −37 °C for PEG_113_–PHBA_260_ (Fig. 2c; purple and red traces, respectively). Interestingly, the melting transition (*T*_m_ = 58 °C^73^) exhibited by the PEG_113_ precursor (Fig. 2c, blue trace) is not observed for either linear diblock copolymer. This is attributed to the ether linkages in the PEG block forming hydrogen bonds with the hydroxyl-functional PHBA or PHPMA blocks. Comparing the DSC traces obtained for the linear PEG_113_–PHBA_260_ (red trace) and GA-crosslinked PEG_113_–PHBA_260_ (green trace) confirmed that such covalent stabilization eliminates the sub-ambient *T*_g_ associated with the PHBA block. Moreover, visual inspection indicated that the crosslinked copolymers formed glassy powders rather than tacky gums. [N. B. the broad feature observed at around 58 °C in the green trace is attributed to the partially crystalline nature of the PEG block, which can no longer form H-bonds with the GA-crosslinked PHBA chains]. FT-IR studies provided further evidence for successful GA crosslinking (Fig. S3†). Spectra recorded for the freeze-dried PEG_113_ precursor, a linear PHBA_200_ homopolymer, a linear PEG_113_–PHBA_600_ diblock copolymer and a GA-crosslinked PEG_113_–PHBA_600_ diblock copolymer are consistent with the reaction of GA with (some of) the hydroxyl groups on the PHBA chains to form ether linkages (Fig. 2b).^72^

In summary, DSC and FT-IR studies suggest that covalent stabilization *via* GA crosslinking should be sufficient to enable good-quality images to be obtained *via* conventional TEM. Indeed, TEM studies of an aqueous dispersion of GA-crosslinked PEG_113_–PHBA_260_ nano-objects prepared at 5% w/w using a [GA]/[HBA] molar ratio of 0.66 indicated the presence of mainly spheres with minor populations of dimers and trimers (Fig. S4a†). In contrast, no TEM images could be obtained for the corresponding linear PEG_113_–PHBA_260_ nano-objects (data not shown). Similarly, TEM analysis of a relatively viscous dispersion of the analogous linear PEG_113_–PHPMA_260_ nano-objects indicated the presence of short worms with a mean aspect (length/width) ratio of 6–7 (Fig. S4b†). This subtle difference in copolymer morphology suggests that the PHBA block is less hydrophobic than the equivalent PHPMA chains of the same mean DP. Thus, a higher PHBA DP is required to achieve the same copolymer morphology.

**Fig. 3:**
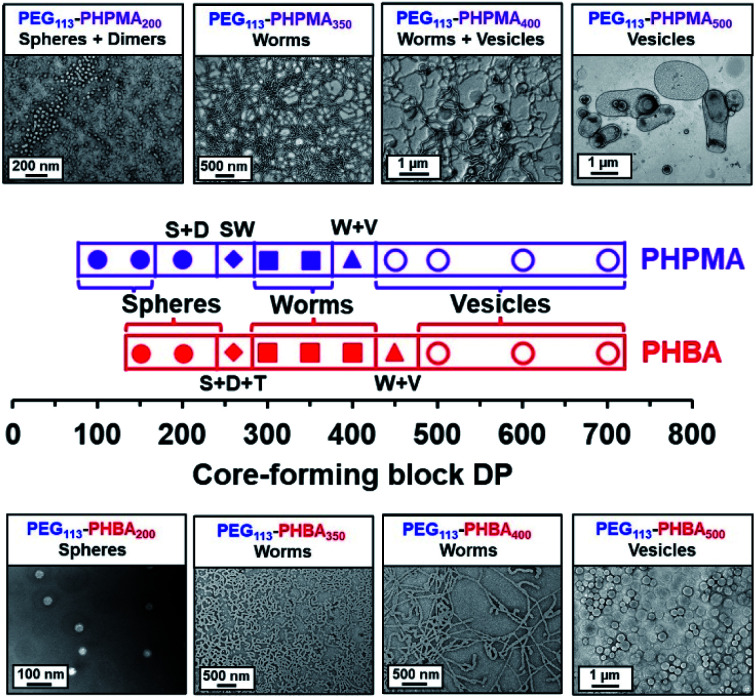
Representative TEM images recorded for two series of PEG_113_–PHBA_*x*_ and PEG_113_–PHPMA_*y*_ diblock copolymer nano-objects prepared *via* RAFT aqueous dispersion polymerization of either HBA or HPMA at 30 °C when targeting 15% w/w solids. For the PEG_113_–PHBA_*x*_ nano-objects, TEM studies required covalent stabilization using a GA crosslinker at 20 °C. Spheres, dimers, trimers, short worms, and worm plus vesicle mixed phases are denoted by S, D, T, SW and W + V, respectively.

Two series of PEG_113_–PHPMA_100–700_ and PEG_113_–PHBA_150–700_ nano-objects were prepared by RAFT dispersion polymerization at 30 °C to enable the copolymer morphology to be directly compared at a given mean DP for the hydrophobic (meth)acrylic block. The PEG_113_–PHBA_100–700_ nano-objects were then crosslinked using GA under the optimized conditions established above. TEM studies of these two copolymer series confirmed that spheres, worms and vesicles could be obtained in both cases, with the less hydrophobic PHBA-based nano-objects typically requiring marginally higher DPs to cross each phase boundary (Fig. 3).

In particular, significantly higher PHBA DPs had to be targeted to form spheres (PHBA DP = 150–200 compared to PHPMA DP = 100–150), which is consistent with the kinetic studies (Fig. S5†). Interestingly, worms were obtained at the same structure-directing DP (DP = 300). However, the more weakly hydrophobic PHBA block provided access to a pure worm phase up to a DP of 400, whereas the corresponding PEG_113_–PHPMA_400_ only produced a mixture of worms and vesicles (Fig. 3). Moreover, pure vesicles were obtained when the PHBA DP was increased up to 500.

**Fig. 4:**
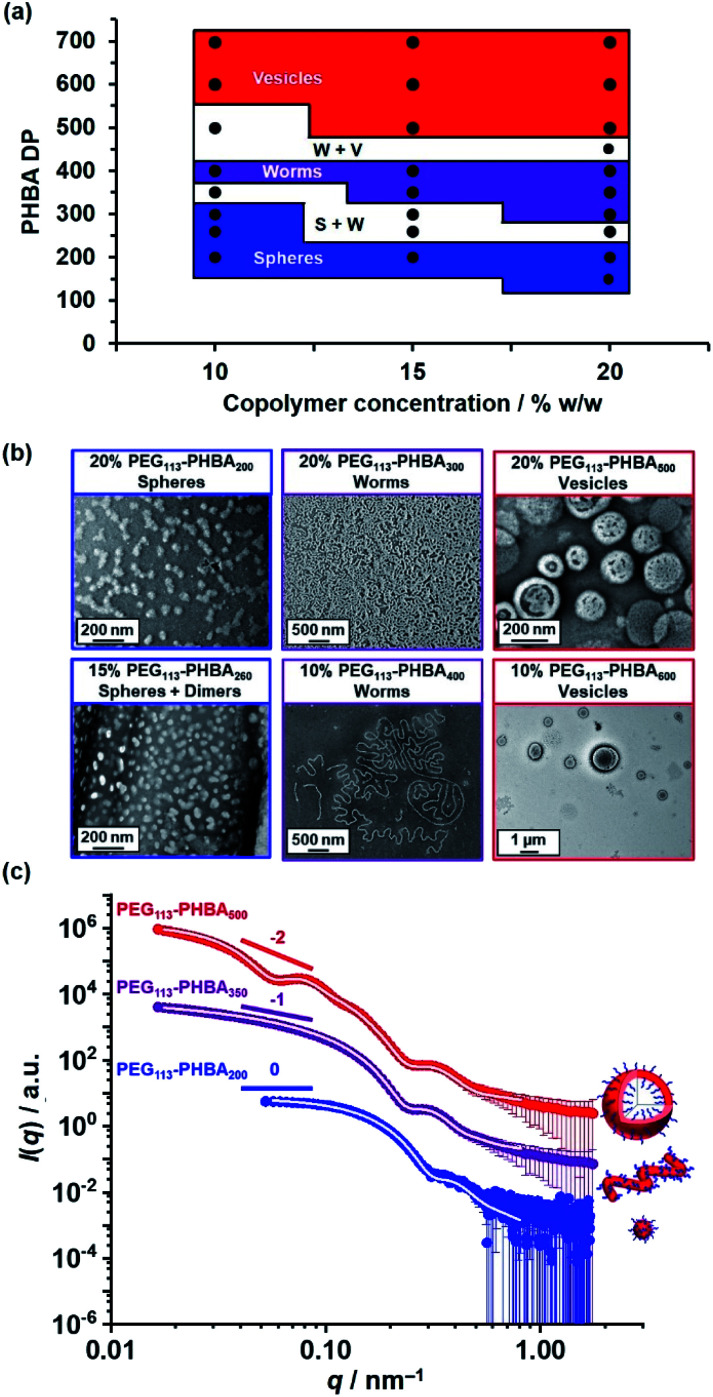
(a) Pseudo-phase diagram constructed for a series of PEG_113_–PHBA_*x*_ nano-objects synthesized *via* RAFT aqueous dispersion polymerization of HBA at 10%, 15% or 20% w/w solids based on a combination of TEM and DLS studies conducted at 20 °C (see Table S1† for the corresponding DLS data). S + W and W + V refer to sphere plus worm and worm plus vesicle mixed phases, respectively. (b) Representative TEM images recorded for a series of GA-crosslinked PEG_113_–PHBA_*x*_ nano-objects after GA crosslinking at 20 °C using a [GA]/[HBA] molar ratio of 0.66. (c) Selected SAXS patterns (black, blue and red symbols) and corresponding data fits (solid white lines) obtained for 1.0% w/w aqueous copolymer dispersions of linear PEG_113_–PHBA_200_ spheres, PEG_113_–PHBA_350_ worms and PEG_113_–PHBA_600_ vesicles at 20 °C and pH 7. See Table S1† for a summary of the nano-object dimensions calculated using the appropriate scattering models.^80,81^

Warren *et al.* reported the formation of PEG_113_–PHPMA_260_ vesicles at 15% w/w solids, rather than the weakly anisotropic PEG_113_–PHPMA_260_ worms shown in Fig. S4b.†^52^ However, the former PISA syntheses were conducted at 50 °C, whereas the latter worms were prepared at 30 °C and it is well known that the precise reaction conditions can significantly influence the final copolymer morphology for this aqueous PISA formulation.^38,52^ Given the numerous studies and various potential applications for thermoresponsive PHPMA-based (and other structure-directing block)^74^ nano-objects already reported in the literature,^75–77^ the current study is focused on understanding the thermoresponsive behavior exhibited by the new PHBA-based nano-objects. Accordingly, a pseudo-phase diagram was constructed for a series of PEG_113_–PHBA_*x*_ nano-objects prepared at 10–20% w/w solids *via* RAFT aqueous dispersion polymerization of HBA at 30 °C (Fig. 4a). GA crosslinking of the PHBA chains under optimized conditions enabled good-quality TEM images to be obtained (Fig. 4b).

For the PDMAC_56_–P(HBA-*stat*-DAAM)_264_ nano-objects recently reported by Byard *et al.*,^50^ the terminal carboxylic acid group located on the PDMAC steric stabilizer chains meant that the solution pH had to be kept relatively low (pH 3) both before and after the PISA synthesis in order to suppress end-group ionization, otherwise only kinetically-trapped spheres were obtained.^78^ In contrast, the non-ionic nature of the PEG_113_ stabilizer block employed in the current study enabled the solution pH to be adjusted up to pH 8 without generating kinetically-trapped spheres, as confirmed by DLS and aqueous electrophoresis studies (Fig. S6†).

Fig. 4c shows the X-ray scattering intensity, *I*(*q*), plotted as a function of the scattering vector, *q*, for 1.0% w/w aqueous dispersions of linear PEG_113_–PHBA_200_ (blue symbols), PEG_113_–PHBA_350_ (purple symbols) and PEG_113_–PHBA_500_ (red symbols) nano-objects at 20 °C and pH 7. Unlike TEM, these SAXS data are averaged over many millions of nanoparticles so they are much more statistically reliable. Moreover, GA crosslinking is not required to stabilize the copolymer morphology prior to SAXS studies. The SAXS pattern recorded for PEG_113_–PHBA_200_ nano-objects (Fig. 4c, blue symbols) had a low *q* gradient close to zero, which is characteristic of spheres. In contrast, the SAXS pattern recorded for PEG_113_–PHBA_350_ (purple symbols) has a low *q* gradient of −1, which is suggests a worm morphology.^52^ Finally, the SAXS pattern obtained for the PEG_113_–PHBA_500_ nano-objects (Fig. 4c, red symbols) had a low *q* gradient of approximately −2, which is consistent with a vesicle morphology.^79^ In each case, the data could be satisfactorily fitted using well-established models for the corresponding copolymer morphology. Moreover, the calculated SAXS dimensions for these linear nano-objects were consistent with TEM data, suggesting that GA crosslinking does not affect the copolymer morphology (Table S2†).^80,81^

**Fig. 5:**
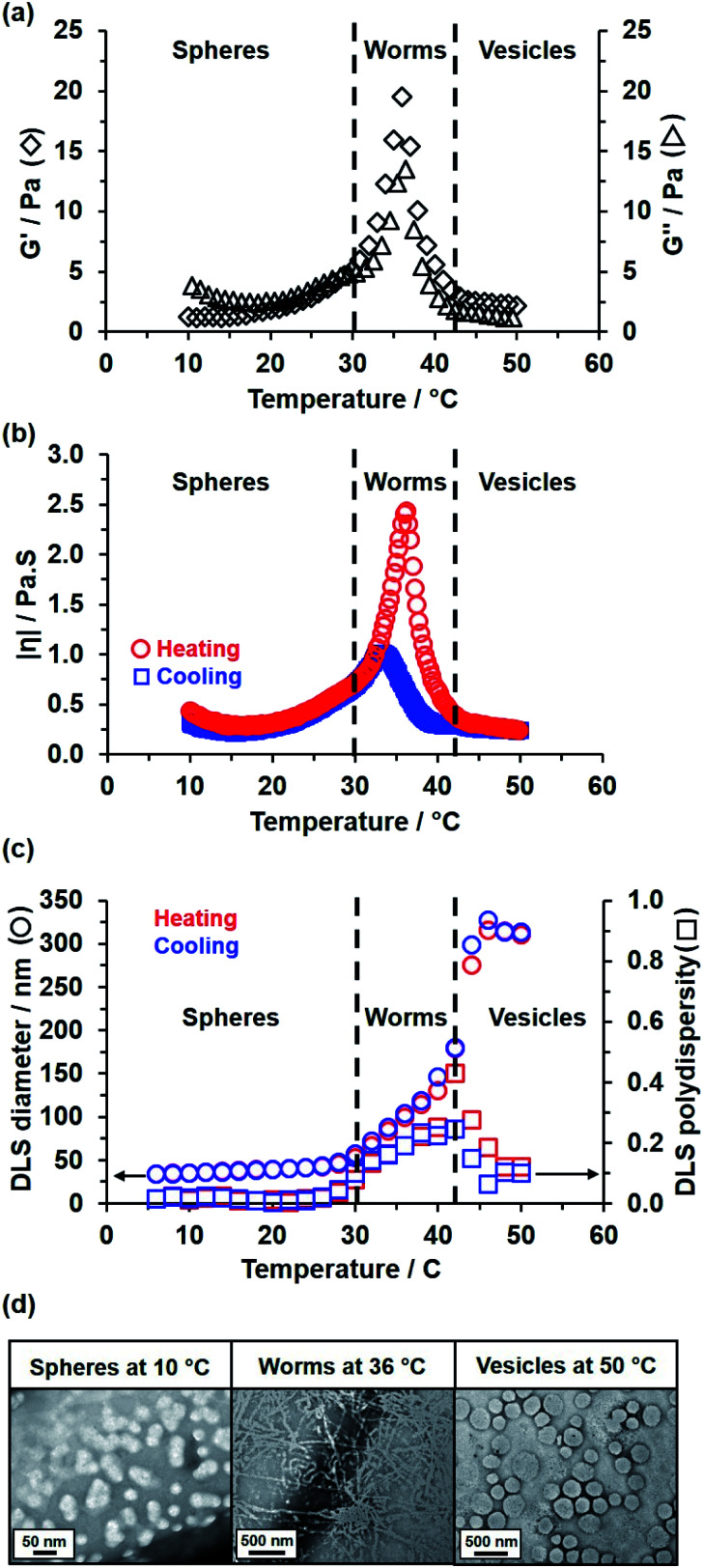
Temperature-dependent rheological studies of a 15% w/w aqueous dispersion of PEG_113_–PHBA_260_ nano-objects as a function of (a) *G*′ (black diamonds) and *G*′′ (black triangles) during a heating cycle and (b) complex viscosity during heating (red circles) and cooling (blue squares) runs. (c) The corresponding variation in *z*-average diameter (circles) and DLS polydispersity (squares) during heating (red) and cooling (blue) runs. The black dashed lines in (a) indicate the sol–gel transitions observed on heating as determined from the *G*′ and *G*′′ values. (d) Representative TEM images recorded for 0.05% w/w PEG_113_–PHBA_260_ nano-objects after their covalent stabilization *via* GA crosslinking conducted at 10, 36 or 50 °C, respectively.

Rheological studies conducted on a 15% w/w aqueous dispersion of linear PEG_113_–PHBA_260_ nano-objects indicated that a low-viscosity fluid is obtained between 10 and 30 °C (Fig. 5a). Heating this dispersion results in the formation of a soft, highly transparent, free-standing gel: the storage modulus (G′) exceeds the loss modulus (G′′) at 30–32 °C and reaches a maximum value of 20 Pa at 36 °C, which corresponds to the formation of highly linear worms with multiple inter-worm contacts.^82,83^ Further heating results in a significant reduction in *G*′ at 42 °C and a concomitant increase in solution turbidity, which is consistent with a worm-to-vesicle transition.^84,85^ As expected, temperature-dependent rheological studies performed on the 15% w/w aqueous dispersion of PEG_113_–PHPMA_260_ short worms confirmed no thermoresponsive behavior between 15 and 55 °C (Fig. S7†).^82,86,87^ The complex viscosity of the 15% w/w aqueous dispersion of linear PEG_113_–PHBA_260_ nano-objects was monitored during a thermal cycle (Fig. 5b). This data set indicated essentially no hysteresis during the sphere-to-worm and worm-to-vesicle transitions. Variable temperature DLS and SAXS studies (Fig. 5c and S8†) conducted on aqueous dispersions of linear PEG_113_–PHBA_260_ nano-objects are fully consistent with the rheology data reported in Fig. 5b (Table S3†). Moreover, SAXS analysis indicated that the solvent volume fraction (*φ*_sol_) of the PHBA chains increases from 0.10 for spheres at 10 °C to 0.49 for worms at 36 °C to 0.68 for vesicles at 50 °C. Finally, the linear PEG_113_–PHBA_300_ nano-objects undergo inter-conversion between spheres, worms and vesicles over a similar temperature range (see later). In contrast, DLS data obtained for GA-crosslinked PEG_113_–PHBA_300_ worms prepared at 20 °C indicated minimal change in the sphere-equivalent hydrodynamic diameter and DLS polydispersity (see Fig. S9†), which confirms successful covalent stabilization.

**Scheme 2:**
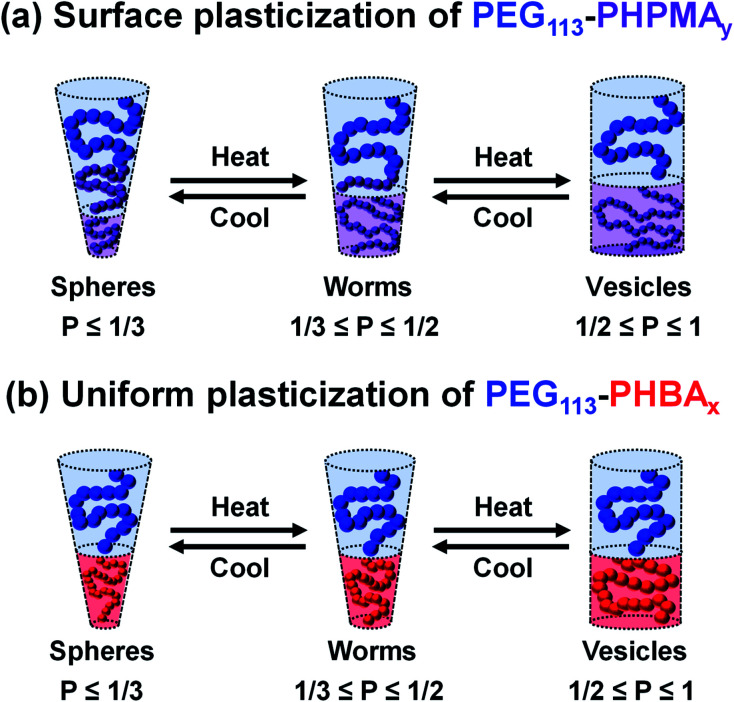
Schematic cartoons indicating (a) the surface plasticization exhibited by thermoresponsive PEG_113_–PHPMA*_y_* nano-objects (note the subtle shift in the effective block junction) and (b) the uniform plasticization exhibited by thermoresponsive PEG_113_–PHBA*_x_* nano-objects. The associated copolymer morphologies and their characteristic packing parameters are also indicated.

It is well-documented that the shape-shifting behavior observed for certain aqueous dispersions of thermoresponsive diblock copolymer nano-objects is the result of a subtle change in the relative degree of hydration of the hydrophobic block (Scheme 2).^84^ For PHPMA-based nano-objects, this is believed to involve hydration of HPMA repeat units located near the block junction (surface plasticization; Scheme 2a). This has been described as an ‘LCST-like’ transition because greater (partial) hydration is observed on lowering the temperature.^28,32,52,53,88,89^ In contrast, HBA-rich chains appear to exhibit ‘UCST-like’ behavior.^50^ This simply requires that the weakly hydrophobic PHBA chains become more solvated on heating while remaining insoluble.

**Fig. 6:**
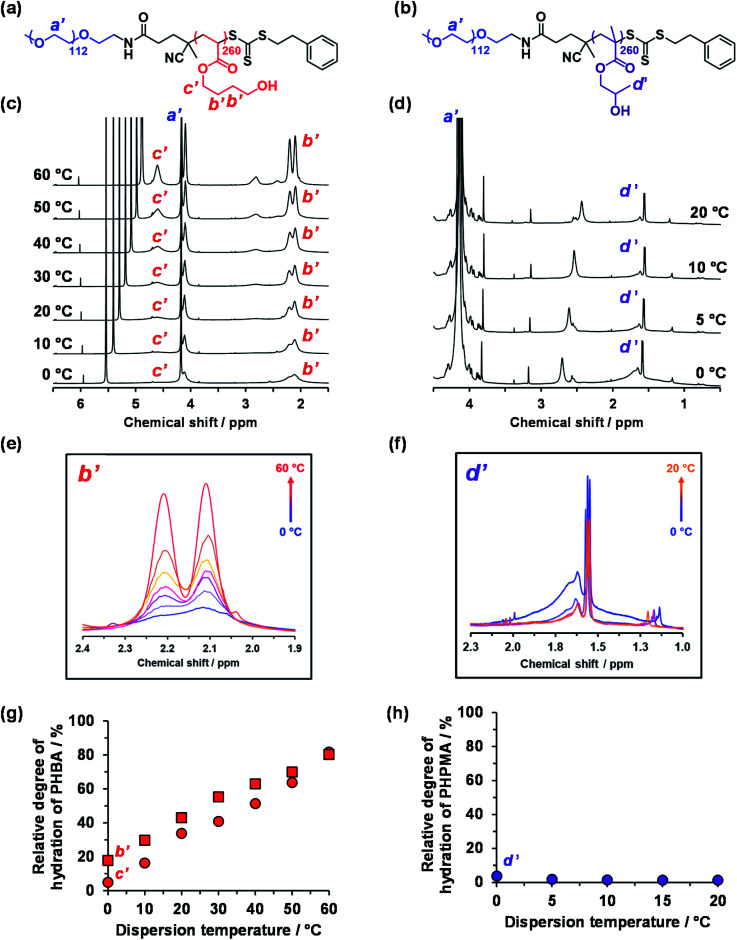
Variable temperature ^1^H NMR studies of thermoresponsive linear PEG_113_-stabilized nano-objects dispersed in D_2_O (see HDO signal at 5.0–5.5 ppm in panel (c)). Chemical structures of the (a) PEG_113_–PHBA_260_ and (b) PEG_113_–PHPMA_260_ diblock copolymers with assignment of the corresponding proton signals. (b) Normalized [relative to an external standard (pyridine in 1,1,2,2-tetrachloroethane-d_2_) at ∼7.2 and 6.0 ppm, respectively] partial ^1^H NMR spectra recorded for a 15% w/w aqueous dispersion of (c) PEG_113_–PHBA_260_ and (d) PEG_113_–PHPMA_260_ nano-objects prepared in D_2_O on heating from 0 °C to 60 °C (N.B. the intensity of the proton signals in (d) did not change after 20 °C and are excluded for clarity). (e) Overlaid partial spectra recorded between 0 °C (blue data) and 60 °C (red data) for the four ethyl protons (b′) at 1.9–2.4 ppm within the HBA repeat units. Clearly, the PHBA block becomes more hydrated at higher temperature. (f) Overlaid partial spectra recorded between 0 °C (blue data) and 20 °C (orange data) for the pendent methyl protons (d′) at 1.3–2.0 ppm within the HPMA repeat units. The PHPMA block is only weakly hydrated at 0 °C and becomes slightly dehydrated at higher temperature. Relative degrees of hydration calculated for the hydrophobic (g) PHBA_260_ and (h) PHPMA_260_ block as a function of temperature when using proton signals a′, b′, c′ and d′. See Fig. S11† for assigned ^1^H NMR spectra for both PEG_113_–PHPMA_300_ and PEG_113_–PHBA_300_ diblock copolymers molecularly dissolved in CD_3_OD.

Variable temperature ^1^H NMR studies conducted between 0 and 60 °C on a 15% w/w aqueous dispersion of linear PEG_113_–PHBA_260_ nano-objects prepared in D_2_O yielded the spectra shown in Fig. 6. Importantly, normalizing the PEG integrals relative to those of the pyridine external standard confirmed that there was only a minimal change (*ca*. 5% reduction) in the degree of hydration of the PEG chains from 0 to 50 °C. Similar observations were reported for aqueous solutions of low molecular weight PEG homopolymer using ultrasonic velocity measurements.^90^ At 0 °C, the c′ signal is barely visible (estimated degree of hydration is ∼5%; see ESI†) but the more prominent b′ protons – which are closer to the terminal hydroxyl group – suggest a significantly higher degree of hydration of 27%. Clearly, the apparent relative degree of hydration calculated for the PHBA block depends on which proton signal is selected for analysis: proton signals arising from pendent hydrophilic groups are more prominent compared to those associated with the hydrophobic acrylic backbone.^91,92^ However, regardless of which proton signal is selected for quantification, the ^1^H NMR spectra shown in Fig. 6 indicate that the PHBA block becomes significantly more hydrated at higher temperature. This is fully consistent with the increase in *φ*_sol_ calculated from variable temperature SAXS experiments (Fig. S8 and Table S3†). Furthermore, variable temperature ^1^H NMR studies of a linear PEG_113_–PHBA_600_ confirm that no discernible hysteresis occurs during a 0 °C to 50 °C to 0 °C thermal cycle (Fig. S10†). Such changes in the (partial) degree of hydration of the PHBA block are sufficient to increase its effective volume fraction relative to that of the PEG block and hence drive an evolution in morphology from spheres to worms to vesicles as a result of the increase in the fractional packing parameter *P* (Scheme 2b). However, unlike the PHPMA-based nano-objects, such shape-shifting behavior must involve uniform plasticization, rather than surface plasticization.^50^

**Fig. 7:**
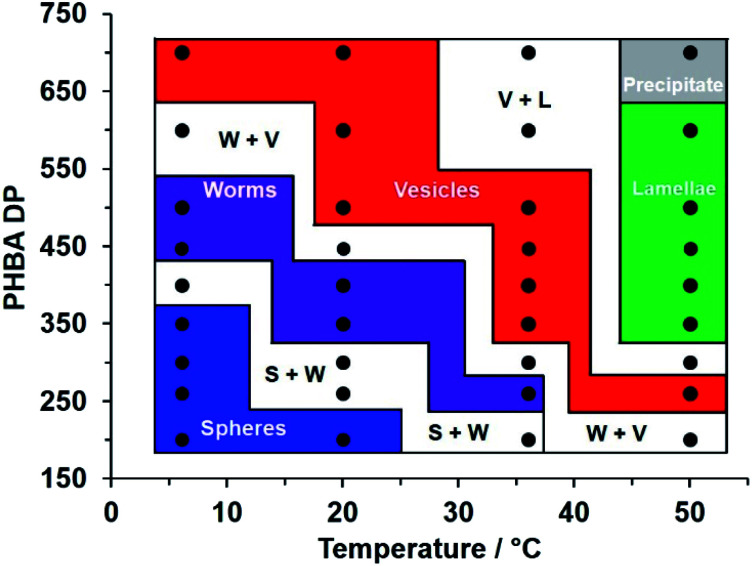
Variable temperature phase diagram constructed for a series of linear PEG_113_–PHBA_*x*_ nano-objects prepared *via* RAFT aqueous dispersion polymerization of HBA targeting 15% w/w solids. Copolymer morphologies were initially assigned on the basis of visual appearance and corroborated by SAXS (Fig. 4c and S8†), TEM (Fig. 3, 4b and 5d) and rheological studies (Fig. 5 and S11†). [N. B. S + W, W + V and V + L denote mixed phases comprising spheres plus worms, worms plus vesicles or vesicles plus lamellae, respectively].

The variable temperature ^1^H NMR spectra recorded between 0 °C and 20 °C for a 15% aqueous dispersion of PEG_113_–PHPMA_260_ nano-objects prepared directly in DCl/D_2_O are shown in Fig. 6d. These spectra are consistent with the variable temperature ^1^H NMR spectra reported by Blanazs *et al.*^7^ and Ratcliffe and co-workers.^84^ However, the choice of PEG as a steric stabilizer block eliminates the problem of overlapping proton signals and so enables quantification for the first time in the present study.

More specifically, the degree of hydration of PHPMA chains is reduced from 4% to 1% on heating from 0 to 20 °C in the present study. However, further heating up to 60 °C led to no further change in the degree of hydration of the PHPMA chains.

This is in striking contrast to the monotonic increase in the (partial) degree of hydration of the PHBA chains observed over the whole temperature range for PEG_113_–PHBA_260_ nano-objects. Given that HPMA and HBA are structural isomers, it is highly counter-intuitive that their corresponding hydroxyl-functional (meth)acrylic blocks should exhibit such qualitatively different temperature-dependent hydration behavior.

Reconstitution of worm gels *via* redispersal of freeze-dried copolymer powder has been demonstrated for PGMA–PHPMA diblock copolymers.^93^ This is an important advantage for potential cell biology applications, because it means that worm gels can be readily prepared *via* copolymer redispersion in the cell culture medium of choice.^7^ TEM analysis of PEG_113_–PHBA_200–700_ nano-objects (after covalent stabilization using the GA crosslinker) confirmed that essentially the same copolymer morphology (spheres, worms or vesicles) could be obtained before and after reconstitution of an aqueous dispersion at a given temperature (Fig. S12†).

Typically, studies of thermoresponsive nano-objects focus on a single diblock copolymer composition.^50,84^ Here, a variable temperature phase diagram was constructed for a series of 15% w/w aqueous dispersions of PEG_113_–PHBA_*x*_ diblock copolymer nano-objects that had been equilibrated for 2 h at 6 °C, 20 °C, 37 °C or 50 °C (Fig. 7). At 20 °C, visual inspection suggested one dispersion comprising spheres (PHBA DP = 200), two mixed sphere/worm phases (PHBA DP = 260–300), two worm dispersions (PHBA DP = 350–400), one mixed worm/vesicle phase and three vesicle dispersions (PHBA DP = 500–700). On cooling these nine aqueous dispersions to 6 °C, visual inspection indicated that the PEG_113_–PHBA_200–350_ nano-objects formed highly transparent free-flowing fluids, with DLS and TEM analysis confirming the presence of spheres. PEG_113_–PHBA_400_ formed a mixed sphere/worm phase, whereas a transparent, free-standing pure worm gel was formed by PEG_113_–PHBA_500_ (complex viscosity = 20 Pa s; Fig. S13†). For *x* > 500, increasingly turbid copolymer dispersions were observed, with a pure vesicle phase being obtained for PEG_113_–PHBA_700_ (Fig. 7). These copolymer dispersions were then assessed at 37 °C. The PEG_113_–PHBA_200_ spheres began to undergo a sphere-to-worm transition at this temperature but only formed a pure worm phase at 44 °C (Fig. S13†). The PEG_113_–PHBA_260_ spheres formed at 20 °C underwent 1D stochastic fusion to generate worms at 37 °C, while the PEG_113_–PHBA_350–400_ worms produced at 20 °C had evolved into vesicles at 37 °C. Finally, the PEG_113_–PHBA_260_ worm gel observed at 37 °C formed a highly turbid dispersion of pure vesicles at 50 °C (Fig. 5d) while the three examples of diblock copolymer vesicles with PHBA DPs = 350–500 were transformed into free-standing turbid pastes comprising lamellae (Fig. S14†).

In summary, PEG_113_–PHBA_*x*_ nano-objects are clearly much more thermoresponsive than their isomeric PEG_113_–PHPMA_*x*_ counterparts and other structure-directing blocks.^42,94^ This observation is expected to be important for the rational design of thermoresponsive diblock copolymer nano-objects for potential biomedical applications.

## Conclusions

The RAFT aqueous dispersion polymerization of HBA using a trithiocarbonate-capped PEG_113_ precursor has been explored for the preparation of concentrated aqueous dispersions of thermoresponsive PHBA-based spheres, worms, vesicles or lamellae. Glutaraldehyde was employed to covalently stabilize these PEG_113_–PHBA_*x*_ nano-objects: successful crosslinking was confirmed by DSC and FT-IR spectroscopy studies and proved to be essential for TEM imaging. This derivatization enabled a direct comparison to be made between two series of PEG_113_–PHBA_*x*_ and PEG_113_–PHPMA_*x*_ nano-objects prepared at 30 °C while targeting 15% w/w solids.

TEM studies confirmed that spheres, worms or vesicles could be obtained for both copolymer series, with the PHBA-based nano-objects typically requiring slightly higher PHBA DPs in order to produce the same nano-object morphology. Such observations are consistent with the appreciably higher aqueous solubility of HBA monomer compared to that of HPMA monomer, which implies that PHBA is more weakly hydrophobic than PHPMA.

The evolution in PEG_113_–PHBA_260_ morphology from spheres to worms to vesicles that occurred on heating proved to be highly reversible on cooling, as judged by rheology, DLS and TEM studies. In contrast, the corresponding PEG_113_–PHPMA_260_ nano-objects did not exhibit any thermoresponsive behavior, because the relatively long PHPMA block is too hydrophobic.

Variable temperature ^1^H NMR studies were conducted to assess the degree of hydration of the PHBA chains compared to PHPMA chains. Despite their isomeric nature, PHBA and PHPMA blocks exhibit complementary thermoresponsive behavior. Thus the PHBA chains within PEG_113_–PHBA_260_ nano-objects become significantly more hydrated on heating (from 5% at 0 °C to 85% at 60 °C), whereas PHPMA chains within PEG_113_–PHPMA_260_ nano-objects become slightly more hydrated at the lower end of the same temperature range. This remarkable difference is highly counter-intuitive and wholly unexpected: it could not be predicted from the chemical structures of HBA and HPMA. Given these ^1^H NMR data, the thermoresponsive behaviour exhibited by PEG_113_–PHBA_260_ nano-objects is best explained in terms of a uniform plasticization mechanism. Moreover, the relatively high chain mobility of the low *T*_g_ PHBA block is responsible for the remarkably good thermoreversibility observed for the PEG_113_–PHBA_*x*_ nano-objects, which exhibit remarkably good thermoreversibility. Furthermore, PEG_113_–PHBA_200–700_ nano-objects can be reconstituted *via* direct dissolution of a freeze-dried powder in aqueous solution. Finally, the thermoreversible behavior of these shape-shifting nano-objects was used to construct a first-of-its-kind variable temperature phase diagram to enable the identification of pure spheres, worms, vesicles and lamellae between 6 and 50 °C.

## Author contributions

O. J. D. and S. P. A. conceived this study. O. J. D. performed all the experiments and analyzed the data. S. P. A. obtained the funding for this research project. O. J. D. and S. P. A. co-wrote the manuscript. J. J. made an important contribution to the SAXS data analysis and modeling.

## Conflicts of interest

There are no conflicts to declare.

## Supplementary Material

SC-012-D1SC05022B-s001
