# Supplementary material for: Shape-shifting thermoreversible diblock copolymer nano-objects via RAFT aqueous dispersion polymerization of 4-hydroxybutyl acrylate
Source: Chem Sci. 2021 Oct 7;12(41):13719–29. doi: 10.1039/d1sc05022b (PMC8549797; doi:10.1039/d1sc05022b)
Supplement: SC-012-D1SC05022B-s001 [file SC-012-D1SC05022B-s001.pdf]

Electronic Supplementary Material (ESI) for Chemical Science.
This journal is © The Royal Society of Chemistry 2021

Supporting Information for:

Shape-Shifting Thermoreversible Diblock Copolymer Nano-Objects via RAFT Aqueous Dispersion Polymerization of 4-Hydroxybutyl Acrylate

Oliver. J. Deane, James Jennings and Steven. P. Armes*

Dainton Building, Department of Chemistry, University of Sheffield, Brook Hill, Sheffield, South Yorkshire, S3 7HF, UK.

Materials and Methods S1

Copolymer Characterization S3

Small Angle X-ray Scattering (SAXS) models S6

Supporting Figures S11

Figure S1. DMF GPC curves obtained for the PEG_{113} precursor, three representative PEG_{113}–$PHBA_x$ diblock copolymers, and a PEG_{113}–$PHPMA_{260}$ diblock copolymer............ S11

Figure S2. DSC curves recorded at a heating rate of 10 °C min^{-1} for $PHBA_{50}$, $PHBA_{100}$ and $PHBA_{200}$ homopolymers S12

Figure S3. FTIR spectra recorded for freeze-dried powders of the PEG_{113} precursor, $PHBA_{200}$ homopolymer, linear PEG_{113}-$PHBA_{600}$ diblock copolymer, and GA-crosslinked PEG_{113}-$PHBA_{600}$ diblock copolymer............ S12

Figure S4. TEM images obtained for GA-crosslinked PEG_{113}–$PHBA_{260}$ spheres and linear PEG_{113}–$PHPMA_{260}$ worms............ S13

Figure S5. Kinetics studies conducted during the RAFT aqueous dispersion polymerization of either HPMA or HBA at 30 °C S13

Table. S1. Target diblock copolymer compositions, DLS diameters and polydispersities, and TEM morphology assignments for the PEG_{113}-$PHBA_x$ diblock copolymer nano-objects............ S14

Figure S6. Z-average diameter vs. pH curves and zeta potential vs pH curves S15

Table S2. Z-average DLS diameters, structural parameters obtained from SAXS analysis, and TEM diameters obtained for PEG_{113}-$PHBA_x$ nano-object............ S16

Figure S7. Temperature-dependent rheological data obtained for a 15% w/w aqueous dispersion of PEG_{113}-$PHPMA_{260}$ short worms S17

Figure S8. SAXS patterns recorded for a 1.0% w/w aqueous dispersion of linear thermoresponsive PEG_{113}–$PHBA_{260}$ nano-objects at 10, 36 and 50°C............ S18

Table S3. Z-average DLS diameters, SAXS structural parameters and TEM diameters obtained for PEG_{113}-$PHBA_{260}$ nano-objects at 10, 36 or 50°C............ S18

Figure S9. Variable temperature DLS studies obtained for GA-crosslinked PEG_{113}-$PHBA_{300}$ worms prepared at 20 °C............ S19

Figure S10. Variable temperature ^{1}H NMR studies of thermoresponsive linear PEG_{113}-$PHBA_{600}$ diblock copolymers S19

Figure S11. Assigned ^{1}H NMR spectra of PEG_{113}-$PHPMA_{300}$ (purple) and PEG_{113}-$PHBA_{300}$ (red) diblock copolymer chains in CD_3OD S20

Figure S12. Representative TEM images obtained for 0.05% w/w aqueous dispersions of reconstituted spheres, worms, and vesicles S20

Figure S13. Temperature-dependent complex viscosity data obtained for 15% w/w aqueous dispersions of PEG_{113}-$PHBA_{500}$, PEG_{113}-$PHBA_{350}$, and PEG_{113}-$PHBA_{200}$ S21

Figure S14. SEM and TEM images for GA-crosslinked PEG_{113}-$PHBA_{500}$ lamellae and a SAXS pattern recorded for linear lamellae prepared at 50°C............ S21

References S22

Material and Methods

4-Hydroxybutyl acrylate (HBA) was kindly donated by BASF SE (Ludwigshafen, Germany) and was purified via extraction using *n*-hexane (twenty times) to remove diacrylate impurities. 2-Hydroxypropyl methacrylate (HPMA) was supplied by GEO Specialty Chemicals (Hythe, UK). According to our prior studies, this structure actually comprises 75% of this monomer, with the remainder being 2-hydroxyisopropyl methacrylate.[1] Trithiocarbonate-based poly(ethylene glycol) (PEG_{113}) macro-CTA was prepared and purified as reported elsewhere.[2] Ascorbic acid (AsAc), potassium persulfate (KPS), *n*-hexane, glutaraldehyde (50% aqueous solution), HCl and NaOH were purchased from Sigma-Aldrich (Dorset, UK) and were used as received. CD_3OD and D_2O were purchased from Goss Scientific Instruments Ltd. (Cheshire, UK). All other solvents were purchased from Fisher Scientific (Loughborough, UK) and were used as received. Deionized water was used for all experiments. Dispersion pH was increased using the appropriate amount of 1.0 M HCl and decreased using 1.0 M NaOH.

Synthesis of PEG_{113}-$PHBA_x$ or PEG_{113}-$PHPMA_y$ nano-objects via RAFT aqueous dispersion polymerization at 30 °C

A typical RAFT dispersion polymerization of PEG_{113} –$PHBA_{260}$ at 15% w/w was conducted as follows. A 14 ml sample vial was charged with PEG_{113} macro-CTA (0.100 g, 18.76 µmol), HBA (0.703 g, 4.88 mmol), KPS (1.01 mg, 3.75 µmol; [PEG_{113}]/[KPS] molar ratio = 5.0). In a separate vial AsAc (0.66 mg, 3.75 µmol; [KPS]/[AsAc] molar ratio = 1.0) and pH 3 water (4.561 g; final target polymer concentration = 15% w/w) were combined. These vials were sealed and then placed in an ice bath and nitrogen was passed over the top of the solution for 30 min. After 30 min, the vial containing HBA, PEG_{113} and KPS was immersed in an oil bath set at 30 °C. Then the AsAc solution was added to this solution via a degassed syringe/needle under a nitrogen atmosphere. The polymerization was allowed to proceed for 2 h before quenching by exposing to air and cooling to room temperature. A monomer conversion of >99% was determined *via* ^{1}H NMR studies [CD_3OD; PEG_{113}-$PHBA_{300}$ δ 1.57-1.81 (PHBA –$C\underline{H}_2C\underline{H}_2$- 4H, tt), 1.83-2.00 (PHBA -C$\underline{H}$-CO- 1H, t), 2.23-2.44 (PHBA -$C\underline{H}_2$-CH-CO- 2H, m), 3.61 (PHBA -$C\underline{H}_2$-OH 2H, t), 3.69 (PEG O-$C\underline{H}_2$-$C\underline{H}_2$- 4H, t), 4.31 (PHBA -O-$C\underline{H}_2$- 2H, t; see Fig. S11). DMF GPC studies indicated an M_n of 53.9 kg mol^{-1} and an M_w/M_n of 1.23 (when calibrated using a series of ten near-monodisperse poly(methyl methacrylate) standards). A series of PEG_{113}-$PHBA_x$ block copolymers were prepared between 10-20% w/w by systematically adjusting the HBA/PEG_{113} molar ratio while maintaining a constant PEG_{113}/KPS molar ratio of 5.0 and adjusting the amount of pH 3 water added. PEG_{113}-$PHPMA_y$ diblock copolymer nano-objects were prepared by simply substituting HBA for its isomer, HPMA, with all masses and volumes remaining the same. [CD_3OD; PEG_{113}-$PHPMA_{300}$ δ 0.83-1.18 (PHPMA –$CH_2C\underline{H}_3$-CO- 3H, d), 1.19-1.32 (PHPMA -COH-$C\underline{H}_3$ 3H, s), 1.83-2.22 (PHPMA -$C\underline{H}_2$-C-CO- 2H, m), 3.69 (PEG O-$C\underline{H}_2$-$C\underline{H}_2$- 4H, t), 3.77-4.10 (PHPMA O-$C\underline{H}_2$-C- 2H, d), see Fig. S11).

Synthesis of $PHBA_{50-200}$ or $PHPMA_{200}$ homopolymers via RAFT solution polymerization in methanol at 44 °C

A typical synthesis of $PHBA_{200}$ via RAFT solution polymerization at 40% w/w was conducted as follows. A 14 ml sample vial was charged with PETTC (0.0050 g, 14.1 μmol), HBA (0.4078 g, 2.82 mmol), VA-044 (0.91 mg, 2.8 μmol, target DP = 200; [PETTC]/[VA-044] molar ratio = 5.0) and methanol (0.6206 g; final target polymer concentration = 40% w/w). This vial was sealed and then placed in an ice bath and charged with nitrogen for 30 min. After 30 min, the vial was immersed in an oil bath set at 30 °C. The polymerization was allowed to proceed for 8 h before quenching by exposing to air while cooling to room temperature. More than 99% HBA conversion was determined *via* ^{1}H NMR studies (CD_3OD). DMF GPC studies indicated an M_n of 36.5 kg mol^{-1} and an M_w/M_n of 1.28. Two further $PHBA_x$ homopolymers (targeting x = 50 or 100) were prepared by adjusting the HBA/PETTC molar ratio while maintaining a constant PETTC/VA-044 molar ratio of 5.0 and adjusting the volume of methanol. DMF GPC analysis of the $PHBA_{50}$ and $PHBA_{100}$ homopolymers indicated M_n = 12.7 kg mol^{-1} and 22.5 kg mol^{-1}, and M_w/M_n = 1.14 and 1.18, respectively. A $PHPMA_{200}$ homopolymer was prepared by simply substituting HBA for its isomer, HPMA, with all masses and volumes remaining the same. GPC analysis indicated M_n = 32.2 kg mol^{-1} and M_w/M_n = 1.18 for this $PHPMA_{200}$ homopolymer (when calibrated using a series of ten near-monodisperse poly(methyl methacrylate) standards). This suggests that DMF is a slightly better solvent for $PHBA_{200}$ than for $PHPMA_{200}$, since the former exhibits a larger hydrodynamic volume.

Covalent Stabilization of Diblock Copolymer Nano-objects Using Glutaraldehyde

A typical protocol used for crosslinking PEG_{113}–$PHBA_{260}$ spheres is as follows. A 15% w/w acidic aqueous dispersion of PEG_{113}–$PHBA_{260}$ spheres (0.2 g) was diluted to 5% w/w using water (0.4 g; PHBA = 455 μmol), adjusted to pH 7 (1 M NaOH) and stirred for 4 h in a 7 ml reaction vial. Glutaraldehyde (GA; 34.1 mg, 341 μmol, GA/PHBA molar ratio = 0.66) was then added and stirred at 20 °C for 16 h. Then an aliquot (0.01 g) was extracted and diluted with water (4.99 g; final target solids concentration = 0.05% w/w) and stirred for 24 h prior to preparation of the corresponding TEM grid (see below for further details). Crosslinking of PEG_{113}–$PHBA_x$ worms and vesicles was also performed at 5% w/w. In order to crosslink PEG_{113}-$PHBA_x$ diblock copolymer nano-objects at a particular temperature, 5% w/w dispersions were either equilibrated at the desired reaction temperature for 1 h prior to GA addition and then allowed to crosslink for 16 h before dilution with water (final target solids concentration = 0.05% w/w) that had been pre-equilibrated at the same temperature.

Copolymer Characterization

^{1}H NMR Spectroscopy. Spectra were recorded in either CD_3OD or D_2O using a 400 MHz Bruker AVANCE-400 spectrometer with 64 scans being averaged per spectrum. The relative degree of (partial) hydration of the PHBA chains was calculated by comparing the

integrated signals assigned to the two COO-C$\underline{H}_2$ protons labeled *c'* and the four C$\underline{H}_2$C$\underline{H}_2$ protons labeled *b'* relative to the four oxyethylene protons labeled *a'* assigned to the PEG_{113} stabilizer block at each temperature (see Figure 6). Thus, a degree of hydration of 100% is calculated in such experiments if the *apparent* diblock composition corresponds to that observed by ^{1}H NMR spectroscopy when employing a good solvent for the PEG and PHBA blocks (e.g. CD_3OD).

Gel Permeation Chromatography. Copolymer molecular weights and dispersities were determined using an Agilent 1260 Infinity GPC system equipped with both refractive index and UV−visible detectors. Two Agilent PLgel 5 µm Mixed-C columns and a guard column were connected in series and maintained at 60 °C. HPLC-grade DMF containing 10 mM LiBr was used as the eluent and the flow rate was set at 1.0 mL min^{-1}. Refractive index detection was used for calculation of molecular weights and dispersities by calibration against a series of ten near-monodisperse poly(methyl methacrylate) standards (with M_n values ranging from 370 to 2,520,000 g mol^{-1}).

Differential Scanning Calorimetry. DSC studies were performed using a TA Instruments Discovery DSC 25 instrument equipped with TZero low-mass aluminum pans and vented lids. Copolymers (and homopolymers) were equilibrated above their T_g for 10 min before performing two consecutive thermal cycles at 10 °C min^{-1}. Two cycles were performed to minimize the thermal history of each sample and ensure removal of any residual solvents. Only data obtained during the second thermal cycle are presented.

Fourier Transform Infrared Spectroscopy. FTIR spectra were recorded using a Thermo-Scientific Nicolet IS10 FT-IR spectrometer equipped with a Golden Gate Diamond ATR accessory. Spectra were recorded for freeze-dried homopolymers and copolymers after drying in a vacuum oven at 30 °C for 3 days. Each spectrum was averaged over 256 scans.

Transmission Electron Microscopy. Unless stated otherwise, as-prepared copolymer dispersions were diluted at 20 °C using acidified deionized water (pH 3) to generate 0.05% w/w aqueous dispersions. Copper/palladium TEM grids (Agar Scientific, UK) were coated in-house to produce thin films of amorphous carbon. These grids were then treated with a plasma glow discharge for 30 s to create a hydrophilic surface. One droplet of an aqueous copolymer dispersion (20 µL; 0.05% w/w) was placed on a freshly-treated grid for 1 min and then blotted with a filter paper to remove excess solution. To stain the deposited nanoparticles, an aqueous solution of uranyl formate (10 µL; 0.75% w/w) was placed on the sample-loaded grid via micropipet for 45 s and then carefully blotted to remove

excess stain. Each grid was then dried using a vacuum hose. Imaging was performed using a Philips CM100 instrument operating at 100 kV and equipped with a Gatan 1k CCD camera.

Dynamic Light Scattering. Measurements were conducted at 25 °C using a Malvern Instruments Zetasizer Nano ZS instrument equipped with a 4 mW He−Ne laser (λ = 633 nm) and an avalanche photodiode detector. Scattered light was detected at 173°. Copolymer dispersions were diluted to 0.10% w/w prior to analysis. Intensity-average hydrodynamic diameters were averaged over three runs and calculated using the Stokes−Einstein equation.

Aqueous Electrophoresis. Zeta potential measurements were performed on 0.1% w/w aqueous copolymer dispersions at 20 °C in the presence of 1 mM KCl using the same Malvern Zetasizer Nano ZS instrument. The initial copolymer dispersion pH was pH 2.5 and was adjusted by addition of small amounts of aqueous 0.1 M NaOH, with 10 min being allowed for equilibrium at each pH. Zeta potentials were calculated from the Henry equation using the Smoluchowski approximation. Hydrodynamic DLS diameters were also recorded during these pH sweep experiments. All data were averaged over three consecutive runs.

Rheology. An AR-G2 rheometer equipped with a variable temperature Peltier plate and a 40 mL 2° aluminum cone was used for all experiments. The dispersion viscosity, loss modulus and storage modulus were measured as a function of applied strain, angular frequency, and temperature to assess the gel strength, gel viscosity and critical gelation temperature. Temperature sweeps were conducted using 20% w/w copolymer dispersions at an applied strain of 1.0% and an angular frequency of 1.0 rad s^{-1}. In these latter experiments, the copolymer dispersion was subjected to a single thermal cycle[4] (heating up to 60°C, followed by cooling to 1°C), and then equilibrated at 1°C for 15 min prior to measurements. To obtain these data, the dispersion was initially cooled to 0°C and held for 60 s prior to heating at 2°C intervals, allowing a thermal equilibration time of 60 s between each measurement.

SAXS Studies. SAXS patterns were recorded for 1.0% w/w aqueous copolymer dispersions at Diamond Light Source (station I22, Didcot, UK) using monochromatic synchrotron X-ray radiation (λ = 0.124 nm, with q ranging from 0.015 to 1.300 nm^{-1}, where $q = 4\pi \sin \theta/\lambda$ is the length of the scattering vector and θ is one-half of the scattering angle) and a 2D Pilatus 2M pixel detector (Dectris, Switzerland). Alternatively, a Xeuss 2.0 (Xenocs) SAXS instrument equipped with a FOX 3D multilayered X-ray mirror, two sets of

scatterless slits for collimation, a hybrid pixel area detector (Pilatus 1M, Dectris) and a liquid gallium MetalJet X-ray source (Excillum, λ = 1.34 Å) was used. In the latter case, SAXS patterns were recorded at a sample-to-detector distance of approximately 1.20 m (calibrated using a silver behenate standard). Glass capillaries of 2.0 mm diameter were used as sample holders. SAXS data were reduced (integration, normalization and absolute intensity calibration using SAXS patterns recorded for deionized water assuming that the differential scattering cross-section of water is 0.0162 cm^{-1}) using Dawn software supplied by Diamond Light Source.[3] For the variable temperature experiments, the sample holders were placed in a HFSX350-CAP temperature-controlled stage (Linkam Scientific, Tadworth, UK) and 10 min was allowed between each measurement to ensure thermal equilibration.

Small Angel X-ray Scattering (SAXS) Models

In general, the intensity of X-rays scattered by a dispersion of nano-objects [usually represented by the scattering cross section per unit sample volume, $\frac{d\Sigma}{d\Omega}(q)$] can be expressed as:

$$\frac{d\Sigma}{d\Omega}(q) = NS(q)\int_0^\infty \ldots \int_0^\infty F(q,r_1,\ldots,r_k)^2 \Psi(r_1,\ldots,r_k)dr_1 \ldots dr_k \qquad \text{S1}$$

where $F(q, r_1, \ldots, r_k)$ is the form factor, $r_1, \ldots, r_k$ is a set of k parameters describing the structural morphology, $\Psi(r_1, \ldots, r_k)$ is the distribution function, S(q) is the structure factor and N is the nano-object number density per unit volume expressed as:

$$N = \frac{\varphi}{\int_0^\infty \ldots \int_0^\infty V(r_1,\ldots,r_k)\Psi(r_1,\ldots,r_k)dr_1 \ldots dr_k} \qquad \text{S2}$$

where $V(r_1, \ldots, r_k)$ is volume of the nano-object and φ is their volume fraction in the dispersion. For all SAXS experiments conducted herein, a dilute copolymer concentration of 1.0 % w/w was utilised. As such, for all analysis and modelling it was assumed that s(q) = 1.

Sphere model. The spherical micelle form factor equation for Equation S1 is given by[4]:

$$F_{sph}(q) = N_s^2\beta_s^2A_s^2(q,R_s) + N_s\beta_c^2F_c(q,R_g) + (q) \qquad \text{S3}$$

Where R_s is the core radius of the spherical micelle, R_g, is the radius of gyration of the PEG_{113} corona block. The core block and the corona block X-ray scattering length contrast is given by $\beta_s = V_s(\xi_s - \xi_{sol})$ and $\beta_s = V_c(\xi_c - \xi_{sol})$, respectively. Here ξ_s, ξ_c and ξ_{sol} are the X-ray scattering length densities of the core-forming block (ξ_{PHBA} = 10.65 × 10^{10} cm^{-2}), the coronal

stabilizer block (ξ_{PEG113} = 11.37 × 10^{10} cm^{-2}) and the solvent (ξ_{sol} = 9.42 × 10^{10} cm^{-2}). V_s and V_c are the volumes of the core-forming block and the coronal stabilizer block, respectively. Using the molecular weights of the PHBA and PEG_{113} blocks and their respective mass densities (ρ_{PHBA} = 1.16 g cm^{-3} and ρ_{PEG113} = 1.23 g cm^{-3}), the individual block volumes can be calculated from $V = \frac{M_{n,pol}}{N_A \rho}$, where $M_{n,pol}$ corresponds to the number-average molecular weight of the block determined by ^{1}H NMR spectroscopy. The sphere form factor amplitude is used for the amplitude of the core self-term:

$$A_c(q,R_s) = \Phi(qR_s) exp\left(- \frac{q^2\sigma^2}{2} \right) \quad \text{S4}$$

Where $\Phi(qR_s) = \frac{3[sin(qR_s) - qR_s cos(qR_s)]}{(qR_s)^3}$. A sigmoidal interface between the two blocks was assumed for the spherical micelle form factor (equation S4). This is described by the exponent term with a width σ accounting for a decaying scattering length density at the micellar interface. This σ value was fixed at 2.2 during fitting. The form factor amplitude of the spherical micelle corona is:

$$A_c(q) = \frac{\int_{R_s}^{R_s + 2s} \mu_c(r) \frac{sin(qr)}{qr} r^2 dr}{\int_{R_s}^{R_s + 2s} \mu_c(r) r^2 dr} exp\left(- \frac{q^2\sigma^2}{2} \right) \quad \text{S5}$$

The radial profile, $\mu_c(r)$, can be expressed by a linear combination of two cubic b splines, with two fitting parameters *s* and *a* corresponding to the width of the profile and the weight coefficient, respectively. This information can be found elsewhere,[5,6] as can the approximate integrated form of Equation S5. The self-correlation term for the corona block is given by the Debye function:

$$F_c(q,R_g) = \frac{2[\exp(- q^2R_g^2) - 1 + q^2R_g^2]}{q^4R_g^2} \quad \text{S6}$$

Where R_g is the radius of gyration of the PEG_{113} coronal block. The aggregation number of the spherical micelle is:

$$N_s = (1 - x_{sol}) \frac{\frac{4}{3}\pi R_s^3}{V_s} \quad \text{S7}$$

Where x_{sol} is the volume fraction of solvent in the PHBA micelle core. An effective structure factor expression proposed for interacting spherical micelles[5] has been used in equation S1:

$$S_s(q) = 1 + \frac{A_{s\_mic}^{av}(q)^2[S_{PY}(q,R_{PY},f_{PY}) - 1]}{F_{s\_mic}(q)} \qquad \text{S8}$$

Herein the form factor of the average radial scattering length density distribution of micelles is used as $A_{s\_mic}^{av}(q) = N_s[\beta_s A_s(q,R_s) + \beta_c A_c(q)]$ and $S_{PY}(q,R_{PY},f_{PY})$ is a hard-sphere interaction structure factor based on the Percus-Yevick approximation,[7] where R_{PY} is the interaction radius and f_{PY} is the hard-sphere volume fraction. A polydispersity for one parameter (R_s) is assumed for the micelle model which is described by a Gaussian distribution. Thus, the polydispersity function in Equation S1 can be replaced as:

$$\Psi(r_1) = \frac{1}{\sqrt{2\pi\sigma_{R_s}^2}} exp\left(- \frac{(r_1 - R_s)^2}{2\sigma_{R_s}^2} \right) \qquad \text{S9}$$

Where σ_{R_s} is the standard deviation for R_s. In accordance with equation S2, the number density per unit volume for the micelle model is expressed as:

$$N = \frac{\varphi}{\int_0^\infty V(r_1)\Psi(r_1)dr_1} \qquad \text{S10}$$

where φ is the total volume fraction of copolymer in the spherical micelles and $V(r_1)$ is the total *volume* of copolymer in a spherical micelle $[V(r_1) = (V_s + V_c)N_s(r_1)]$.

Worm-like micelle model

The worm-like micelle form factor in Equation S1 is expressed as[4]:

$$F_{w_{mic}}(q) = N_w^2\beta_s^2 F_w^2(q) + N_w\beta_c^2F_c(q,R_g) + N_w(N_w - 1)\beta_c^2S_{cc}(q) + 2N_w^2\beta_s\beta_cS_{sc}(q) \qquad \text{S11}$$

where all the parameters are the same as in the spherical micelles model (Equation S3). The self-correlation time for the worm-like micelle core or radius is:

$$F_w(q) = F_{worm}(q,L_w,b_w)A_{csworm}^2(q,R_w) \qquad \text{S12}$$

where

$$A_{csworm}^2(q,R_w) = \left[2\frac{J_1(qR_w)}{qR_w}\right]^2 \qquad \text{S13}$$

and J_1 is the first-order Bessel function of the first kind, and a form factor $F_{worm}(q, L_w, b_w)$ for self-avoiding semi-flexible chains represent the worm-like micelle, where b_w is the worm Kuhn length and L_w is the mean worm contour length. A complete expression for the chain form factor can be found elsewhere.[8] The self-correlation term for the corona block is given

by the Debye function shown in Equation S6. The mean aggregation number of the worm-like micelle is given by:

$$N_w = (1 - x_{sol})\frac{\pi R_w^2 L_w}{V_s} \quad \text{S14}$$

where x_{sol} is the volume fraction of solvent within the worm-like micelle core. Possible semi-spherical caps at the ends of each worm are ignored in this form factor. The R_g obtained for the PEG_{113} coronal block of 2.7 nm is comparable to the estimated value of 2.6 nm.

Vesicle model

The vesicle form factor in Equation (S1) is expressed as:

$$F_{ves}(q) = N_v^2\beta_m^2A_m^2(q) + N_v\beta_{vc}^2F_c(q,R_g) + N_v(N_v - 1)\beta_{vc}^2A_{vc}^2(q) + 2N_v^2\beta_m\beta_{vc}A_m(q)A_{vc}(q) \quad \text{S15}$$

The X-ray scattering length contrast for the membrane-forming block (PHBA) and the coronal stabilizer block (PEG_{113}) is given by $\beta_m = V_m(\xi_m - \xi_{sol})$ and $\beta_{vc} = V_{vc}(\xi_{vc} - \xi_{sol})$, respectively, where ξ_m, ξ_{vc} and ξ_{sol} are the X-ray scattering length densities of the membrane-forming block (ξ_{PHBA} = 10.65 × 10^{10} cm^{-2}), the coronal stabilizer block (ξ_{PEG113} = 11.37 × 10^{10} cm^{-2}) and the solvent (ξ_{sol} = 9.42× 10^{10} cm^{-2}). V_m and V_{vc} are the volumes of the membrane-forming block and the coronal stabilizer block, respectively. Using the molecular weights of the PHBA and PEG_{113} blocks and their respective mass densities (ρ_{PHBA} = 1.16 g cm^{-3} and ρ_{PEG113} = 1.23 g cm^{-3}), the individual block volumes can be calculated from $V = \frac{M_{n,pol}}{N_A\rho}$, where $M_{n,pol}$ corresponds to the number-average molecular weight of the block determined by ^1H NMR spectroscopy. The amplitude of the membrane self-term is:

$$A_m(q) = \frac{V_{out}\varphi(qR_{out}) - V_{in}\varphi(qR_{in})}{V_{out} - V_{in}} e^{\left(-\frac{q^2\sigma_{in}^2}{2}\right)} \quad \text{S16}$$

where $R_{in} = R_m - \frac{1}{2}T_m$ is the inner radius of the membrane, $R_{out} = R_m + \frac{1}{2}T_m$ is the outer radius of the membrane, $V_{in} = \frac{4}{3}\pi R_{in}^3$, $V_{out} = \frac{4}{3}\pi R_{out}^3$. It should be noted that Equation S16 differs from that reported in the original work. More specifically, the exponent term in Equation S16 represents a sigmoidal interface between the blocks, with a width σ_{in} accounting for a decaying scattering length density at the membrane surface. The numerical value of σ_{in} was fixed at 2.2. The mean vesicle aggregation number, N_v, is given by:

$$N_v = (1 - x_{sol})\frac{V_{out} - V_{in}}{V_m} \quad \text{S17}$$

where x_{sol} is the solvent (i.e. water) volume fraction within the vesicle membrane. A simpler expression for the corona self-term of the vesicle model than that used for the spherical micelle corona self-term was preferred because the contribution to the scattering intensity from the corona block is much less than that from the membrane block in this case. Assuming that there is no penetration of the solvophilic coronal blocks into the solvophobic membrane, the amplitude of the vesicle corona self-term is expressed as:

$$A_{vc}(q) = \Psi(qR_g)\frac{1}{2}\left[\frac{sin[q(R_{out}+R_g)]}{q(R_{out}+R_g)} + \frac{sin[q(R_{in}-R_g)]}{q(R_{in}-R_g)}\right] \qquad \text{S18}$$

where the term outside the square brackets is the factor amplitude of the corona block copolymer chain such that:

$$\Psi(qR_g) = \frac{1 - exp^{(-qR_g)}}{(qR_g)^2} \qquad \text{S19}$$

The mean experimental R_g value of 2.7 nm for the PEG_{113} coronal block is close to the theoretical value (2.6 nm). The latter can be calculated from the contour length of the PEG_{113} block, L_{PEG113} = 113 × 0.37 nm = 41.8 nm (the projected contour length of an ethylene glycol repeat unit (0.37 nm) was based on a known literature value obtained for the crystal structure of PEG homopolymer).[9] Assuming a PEG Kuhn length of 1 nm,[10] an approximate R_g of $(41.8 \times 1/6)^{0.5}$ = 2.6 nm was calculated.

For the vesicle model, it was assumed that two parameters are polydisperse: the overall radius of the vesicles and the membrane thickness (R_m and T_m, respectively). Each is assumed to have a Gaussian distribution, so the polydispersity function in Equation (S1) can be expressed as:

$$\Psi(r_1,r_2) = \frac{1}{\sqrt{2\pi\sigma_{R_m}^2}} exp^{\left(-\frac{(r_1 - R_m)^2}{2\sigma_{R_m}^2}\right)} \frac{1}{\sqrt{2\pi\sigma_{T_m}^2}} exp^{\left(-\frac{(r_1 - T_m)^2}{2\sigma_{T_m}^2}\right)} \qquad \text{S20}$$

where σ_{Rm} and σ_{Tm} are the standard deviations for R_m and T_m, respectively. Following Equation S2, the number density per unit volume for the vesicle model is expressed as:

$$N = \frac{\varphi}{\int_0^\infty \int_0^\infty V(r_1,r_2)\Psi(r_1,r_2)dr_1 dr_2}$$

S21

where φ is the total volume fraction of copolymer in the vesicles and $V(r_1, r_2)$ is the total volume of copolymers in a vesicle $[V(r_1, r_2) = (V_m + V_{vc})N_v(r_1, r_2)]$.

Programming tools within the Irena SAS Igor Pro macros were used to implement the scattering models.[11]

Supporting Figures

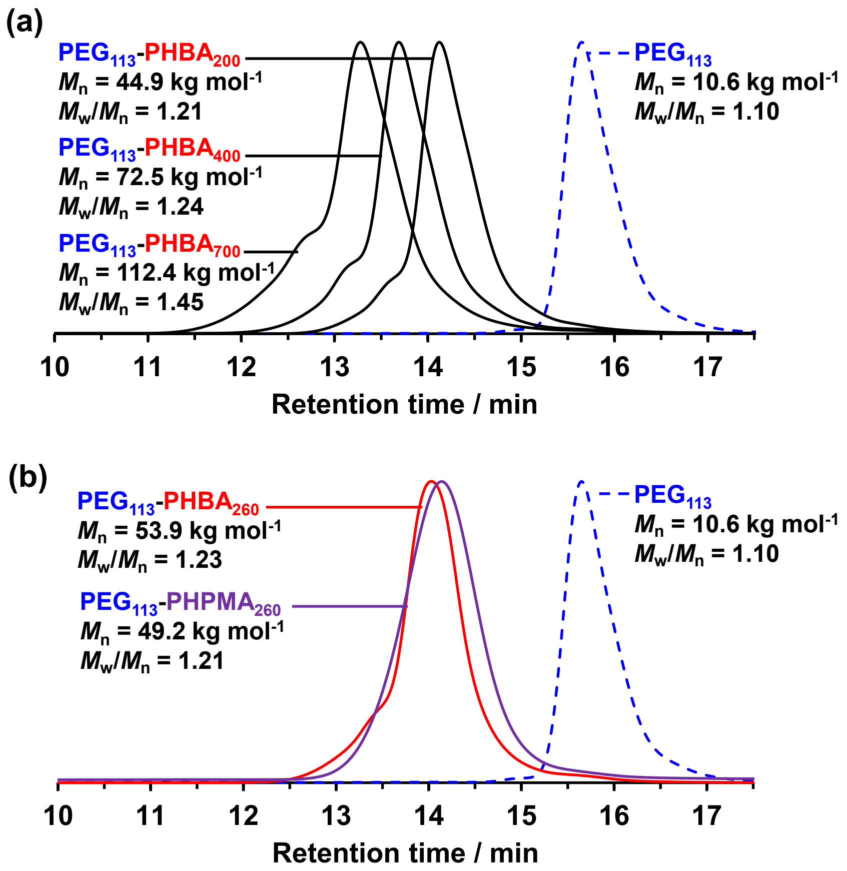

Figure S1. DMF GPC data (expressed relative to a series of poly(methyl methacrylate) calibration standards) obtained for (a) the PEG_{113} precursor and three representative PEG_{113}–$PHBA_x$ diblock copolymers (where x = 200, 400 or 700). The resulting GPC traces are clearly shifted to lower retention times (i.e. higher molecular weights) relative to that of the PEG_{113} precursor (M_n = 10.6 kg mol^{-1}; M_w/M_n = 1.10; when compared to a series of poly(methyl methacrylate) calibration standards). Increasing the target PHBA DP up to 700 resulted in a significant increase in dispersity (M_w/M_n = 1.45) and the appearance of a high molecular weight shoulder. The latter feature suggests that some degree of chain transfer to polymer occurs at 30°C, which is a well-known problem for acrylic monomers.[12–14] (b) the same PEG_{113} precursor, PEG_{113}–$PHBA_{260}$ diblock copolymer, and a PEG_{113}–$PHPMA_{260}$

diblock copolymer used for a direct comparison of the thermoresponsive behavior of these two systems.

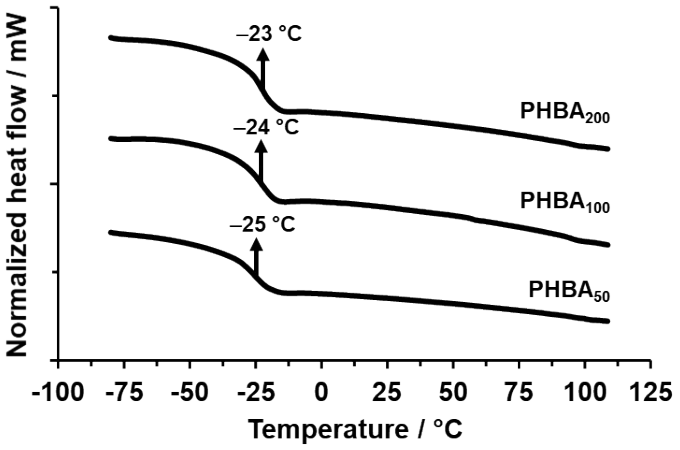

Figure S2. DSC curves recorded at a heating rate of 10 °C min^{-1} for $PHBA_{50}$, $PHBA_{100}$ and $PHBA_{200}$ homopolymers prepared via RAFT solution polymerization of HBA in methanol. Each sample was purified and subsequently dried at 30 °C under vacuum for 3 days prior to analysis. DSC traces are arbitrarily offset for clarity. The data indicate a rather weak relationship between the PHBA T_g and its molecular weight.

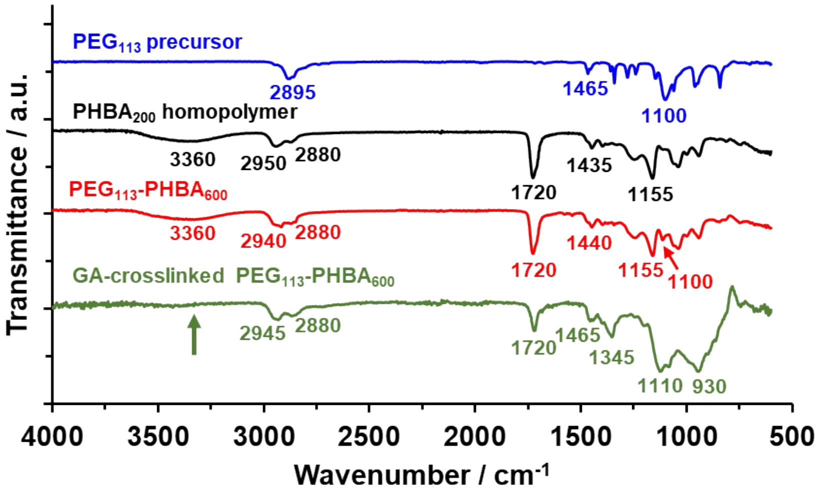

Figure S3. FTIR spectra recorded for freeze-dried powders of the (blue) PEG_{113} precursor, (black) $PHBA_{200}$ homopolymer, (red) linear PEG_{113}-$PHBA_{600}$ diblock copolymer, and (green) GA-crosslinked PEG_{113}-$PHBA_{600}$ diblock copolymer. Core-crosslinking was performed using a [GA]/[HBA] molar ratio of 0.66 at 20 °C for 4 h. Spectra are arbitrarily offset for the sake of clarity. The 2875 cm^{-1} band observed for the PEG_{113} precursor and $PHBA_{200}$ homopolymer is characteristic of the aliphatic C-H stretch (see blue and black traces).[15] The broad band at around 3360 cm^{-1} is assigned to the OH stretch associated with the pendent hydroxyl groups in the HBA repeat units of the linear PEG_{113}-$PHBA_{600}$ diblock copolymer (see red trace).[16–20] Notably, this feature is much less intense in the FT-IR spectrum recorded for GA-crosslinked PEG_{113}-$PHBA_{600}$ (see green trace). Literature precedent suggests that the new band at 1110 cm^{-1} (and most likely also the 1345 cm^{-1} band) is the result of acetal formation (see green trace).[21] Finally, the characteristic strong carbonyl stretch for GA at 1635 cm^{-1} (not shown above)

is not observed in the FT-IR spectrum recorded for the final GA-crosslinked PEG_{113}-$PHBA_{600}$ vesicles, indicating minimal contamination by residual GA.

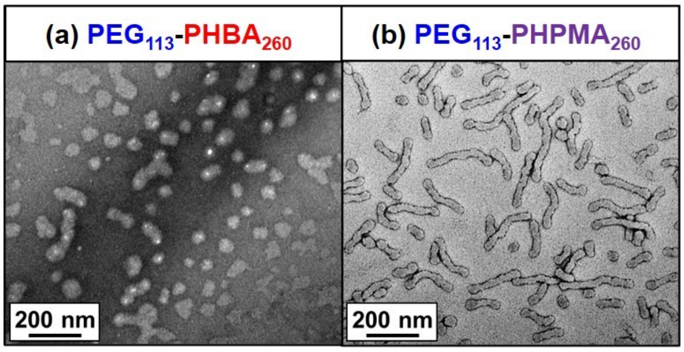

Figure S4. TEM images obtained for (a) PEG_{113}–$PHBA_{260}$ spheres (plus a few short worms) crosslinked with GA at 20°C and (b) linear PEG_{113}–$PHPMA_{260}$ worms (plus a minor population of spheres).

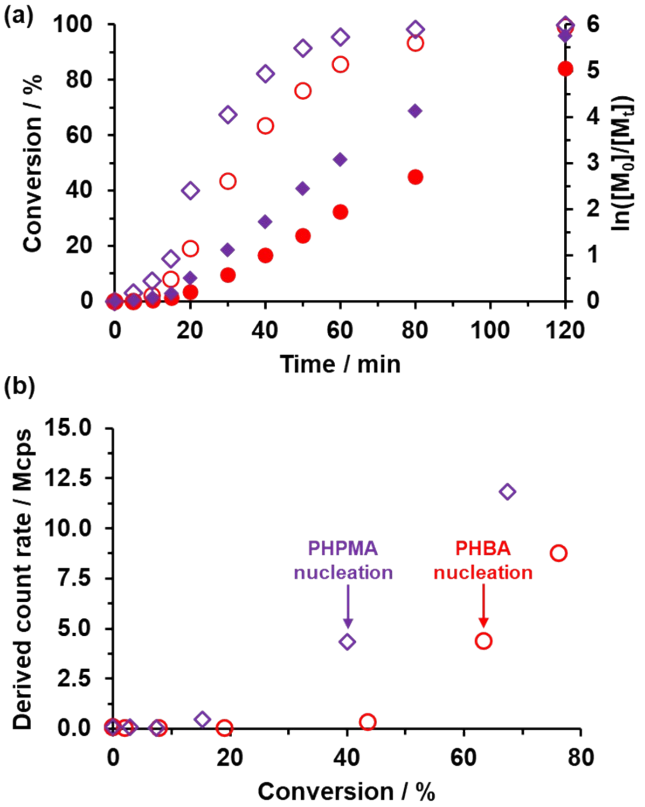

Figure S5. Kinetics studies conducted during the RAFT aqueous dispersion polymerization of either HPMA (purple diamonds) or HBA (red circles) at 30 °C when targeting a core-forming DP of 260 in each case. Conditions: 20% w/w target copolymer concentration, $[PEG_{113}]/[KPS]$ molar ratio = 5.0, pH 3. (a) Conversion (open symbols) *vs* time curves and their corresponding semilogarithmic plots (filled symbols) were determined by ^{1}H NMR spectroscopy. (b) The evolution of derived counts against conversion for either PEG_{113}-$PHPMA_{260}$ (open purple diamonds) or PEG_{113}-$PHBA_{260}$ (open red circles) was determined by DLS studies. The more hydrophobic PHPMA-based nano-objects nucleate at a lower critical monomer conversion (40%) compared to PHBA-based nano-objects (64%), as indicated by

the increase in derived counts from essentially zero (indicating the presence of dissolved copolymer chains) to more than 5 Mcps. In both cases, this increase in the scattered light intensity coincides with an increase in dispersion turbidity, indicating nanoparticle formation.

Table S1. Summary of the target diblock copolymer compositions, DLS particle diameters and polydispersities, and TEM morphology assignments for the PEG_{113}-$PHBA_x$ diblock copolymer nano-objects used to construct the phase diagram shown in Figure 4. [N.B. All TEM studies were conducted on glutaraldehyde-crosslinked nano-objects].

		DLS	TEM
**Diblock copolymer composition**	**Target solids concentration / % w/w**	**Z-average diameter / nm (PDI)**	**Assigned morphology**
PEG_{113}-$PHBA_{200}$	10	42 (0.09)	Spheres
PEG_{113}-$PHBA_{260}$	10	49 (0.05)	Spheres
PEG_{113}-$PHBA_{300}$	10	58 (0.09)	Spheres
PEG_{113}-$PHBA_{350}$	10	154 (0.23)	Spheres + Worms
PEG_{113}-$PHBA_{400}$	10	338 (0.24)	Worms
PEG_{113}-$PHBA_{500}$	10	366 (0.29)	Worms + Vesicles
PEG_{113}-$PHBA_{600}$	10	296 (0.61)	Vesicles
PEG_{113}-$PHBA_{700}$	10	793 (0.54)	Vesicles
PEG_{113}-$PHBA_{200}$	15	41 (0.04)	Spheres
PEG_{113}-$PHBA_{260}$	15	48 (0.12)	Spheres + Dimers
PEG_{113}-$PHBA_{300}$	15	126 (0.17)	Spheres + Worms
PEG_{113}-$PHBA_{350}$	15	155 (0.25)	Worms
PEG_{113}-$PHBA_{400}$	15	414 (0.37)	Worms
PEG_{113}-$PHBA_{500}$	15	534 (0.68)	Vesicles
PEG_{113}-$PHBA_{600}$	15	535 (0.71)	Vesicles
PEG_{113}-$PHBA_{700}$	15	628 (0.54)	Vesicles
PEG_{113}-$PHBA_{150}$	20	38 (0.02)	Spheres
PEG_{113}-$PHBA_{200}$	20	46 (0.05)	Spheres
PEG_{113}-$PHBA_{260}$	20	49 (0.09)	Spheres + Worms
PEG_{113}-$PHBA_{300}$	20	118 (0.20)	Worms
PEG_{113}-$PHBA_{350}$	20	200 (0.22)	Worms
PEG_{113}-$PHBA_{400}$	20	646 (0.43)	Worms
PEG_{113}-$PHBA_{450}$	20	444 (0.18)	Worms + Vesicles
PEG_{113}-$PHBA_{500}$	20	153 (0.08)	Vesicles
PEG_{113}-$PHBA_{600}$	20	373 (0.48)	Vesicles
PEG_{113}-$PHBA_{700}$	20	890 (0.46)	Vesicles

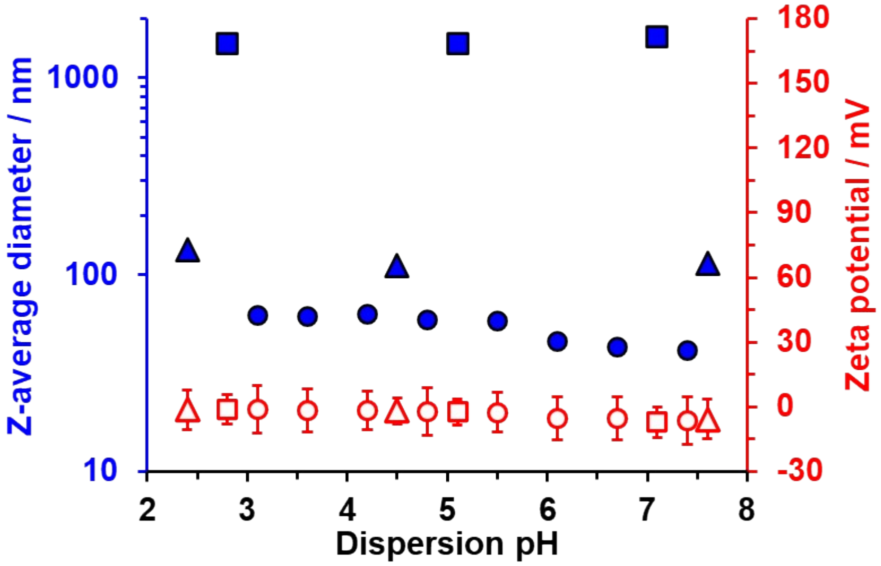

Figure S6. Z-average diameter (blue filled symbols) vs. pH curves and zeta potential (red open symbols) vs pH curves obtained for (a) PEG_{113}-$PHBA_{260}$ spheres (circles), (b) PEG_{113}-$PHBA_{350}$ worms (triangles) and (c) PEG_{113}-$PHBA_{600}$ vesicles (squares). Zeta potentials were determined at 20 °C for 0.1% w/w aqueous copolymer dispersions in the presence of 1 mM KCl. The aqueous dispersion pH was adjusted using either 0.1 M or 1 M NaOH.

Table S2. Summary of z-average DLS diameters, structural parameters obtained from SAXS analysis, and number-average TEM diameters obtained for PEG_{113}-$PHBA_x$ nano-objects at 20°C. DLS studies were conducted on 0.1% w/w aqueous copolymer dispersions (originally synthesized at 20% w/w). SAXS studies were performed on 1.0% w/w aqueous dispersions using well-known sphere, worm or vesicle models for data fits (see the SAXS models section in the supporting information for more information). TEM analysis was conducted on 0.05% w/w aqueous dispersions of glutaraldehyde-crosslinked PEG_{113}-$PHBA_x$ nano-objects. Crosslinking conditions: GA/HBA molar ratio = 0.66, [copolymer] = 5% w/w, 20°C, pH 7.

Diblock copolymer morphology	DLS	SAXS				TEM
	Diameter / nm	Diameter / nm	Worm thickness / nm	Membrane thickness / nm	φ_{sol}	Diameter / nm
PEG_{113}-$PHBA_{200}$ Spheres	44 ± 6	35 ± 4	--	--	0.30	38 ± 5
PEG_{113}-$PHBA_{350}$ Worms	157 ± 97	--	41 ± 5	--	0.30	40 ± 8
PEG_{113}-$PHBA_{500}$ Vesicles	153 ± 48	143 ± 21	--	23 ± 3	0.30	172 ± 56

SAXS analysis of linear **PEG_{113}-$PHBA_{200}$** spheres using a well-established spherical micelle model[4] indicated a volume-average diameter of 35 ± 4 nm for the PHBA cores, which is consistent with an overall hydrodynamic z-average diameter of 44 ± 6 nm reported by DLS (and an estimated TEM number-average diameter of 38 ± 5 nm for the corresponding GA-crosslinked spheres). The SAXS pattern recorded for **PEG_{113}-$PHBA_{350}$** nano-objects was fitted using Pedersen's worm-like micelle model.[4] The worm core cross-sectional diameter was calculated to be 41 ± 5 nm, which is slightly lower than estimated from TEM studies of the GA-crosslinked worms (40 ± 8 nm). However, this is not unreasonable given the additional mass conferred by the GA crosslinker. Moreover, although covalent stabilization is essential for visualization of the copolymer morphology, some degree of worm deformation (flattening) may occur on drying, which would lead to a slightly larger apparent worm core diameter. Unfortunately, the *q* range used for these SAXS studies was too short to enable determination of the overall worm contour length. TEM studies indicated highly linear PEG_{113}-$PHBA_{300-400}$ worms with a broad distribution of worm lengths (0.5-10 μm). The SAXS pattern obtained for the **PEG_{113}-$PHBA_{500}$** nano-objects could be satisfactorily fitted using a well-known vesicle model.[8] This indicated relatively small vesicles with an overall volume-average diameter of 143 ± 21 nm and a mean vesicle membrane thickness of 23 ± 3 nm. This membrane thickness is smaller than both the sphere diameter and worm thickness, which suggests strong interdigitation of the PHBA chains.[22,23] As expected, the above vesicle diameter is lower than the z-average diameter of 153 ± 48 nm reported by DLS. However, TEM studies of the corresponding GA-crosslinked vesicles indicated a slightly higher number-average diameter (172 ± 56 nm), which suggests that some degree of deformation occurs during grid preparation.

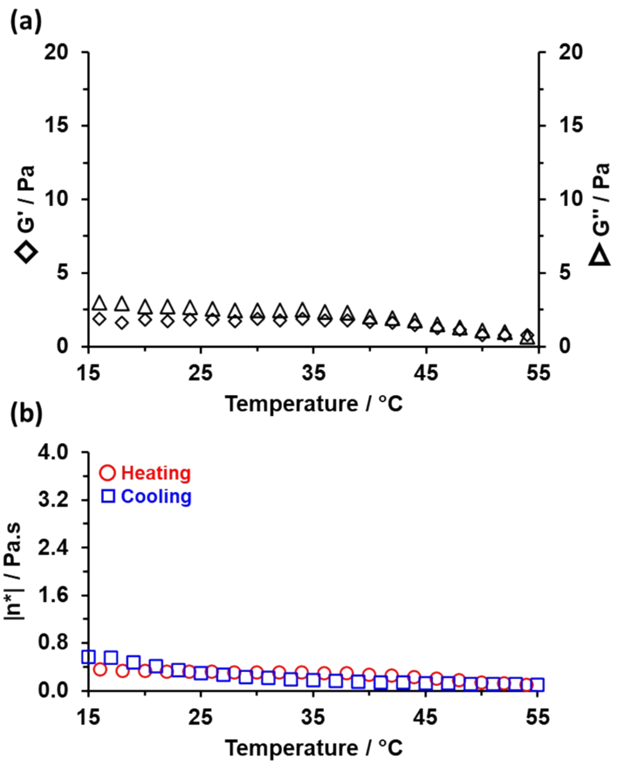

Figure S7. Temperature-dependent rheological data obtained for a 15% w/w aqueous dispersion of PEG_{113}-$PHPMA_{260}$ short worms at an applied strain of 1.0% and an angular frequency of 1.0 rad s^{-1}. This dispersion was held at 5 °C for 30 min prior to heating to ensure thermal equilibration. (a) G' (black diamonds) and G'' (black triangles) and (b) complex viscosity during initial heating (red circles) and subsequent cooling (blue squares) runs. The higher hydrophobicity of the PHPMA-cores inhibited any thermoresponsive behavior. PEG_{113}-$PHPMA_{260}$ short worms indicated no thermoresponsive behavior: this dispersion always remained a viscous fluid (G' = 2.0 Pa and G'' = 2.5 Pa) and never formed a free-standing gel. This is consistent with observations made by Lovett *et al.*[24] and Warren *et al.*,[25] who found that PHPMA-based nano-objects exhibited little or no thermoresponsive behavior if the PHPMA DP exceeded 200.

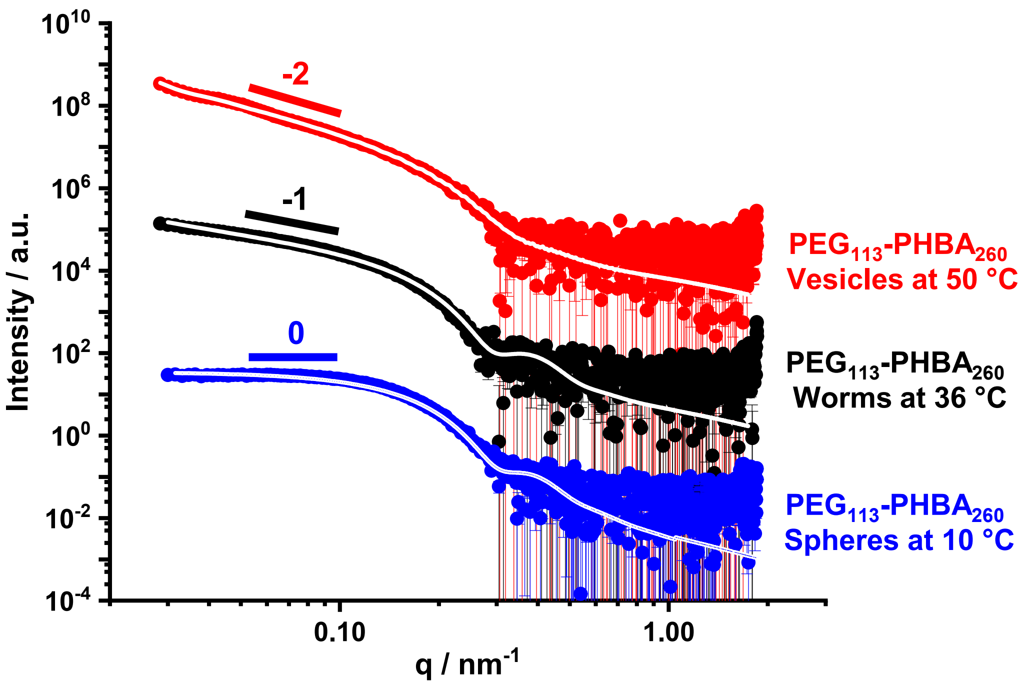

Figure S8. SAXS patterns recorded for a 1.0% w/w aqueous dispersion of linear thermoresponsive PEG_{113}–$PHBA_{260}$ nano-objects at 10°C (blue data), 36°C (black data), 50°C (red data). The white lines indicate the data fits obtained using appropriate scattering models (see Supporting Information for further details).[4,8]

Table S3. Summary of z-average DLS diameters, structural parameters calculated from SAXS analysis and number-average TEM diameters obtained for PEG_{113}-$PHBA_{260}$ nano-objects at 10, 36 or 50°C. DLS studies were conducted using 0.1% w/w aqueous dispersions. SAXS studies were performed on 1.0% w/w aqueous dispersions using well-known sphere, worm or vesicle models for data fits (see the SAXS models section in the supporting information for more information). TEM analysis was conducted using 0.05% w/w aqueous dispersions of GA-crosslinked PEG_{113}-$PHBA_x$ nano-objects. Crosslinking conditions: GA/HBA molar ratio = 0.66, [copolymer] = 5% w/w, pH 7.

PEG_{113}-$PHBA_{260}$ diblock copolymer morphology	Temp. / °C	DLS	SAXS				TEM
		Diameter / nm	Diameter / nm	Worm thickness / nm	Membrane thickness / nm	φ_{sol}	Diameter / nm
Spheres	10	41 ± 5	35 ± 5	--	--	0.10	38 ± 5
Worms	36	179 ± 115	--	32 ± 4	--	0.49	40 ± 8
Vesicles	50	230 ± 110	263 ± 55	--	16 ± 3	0.68	271 ± 69

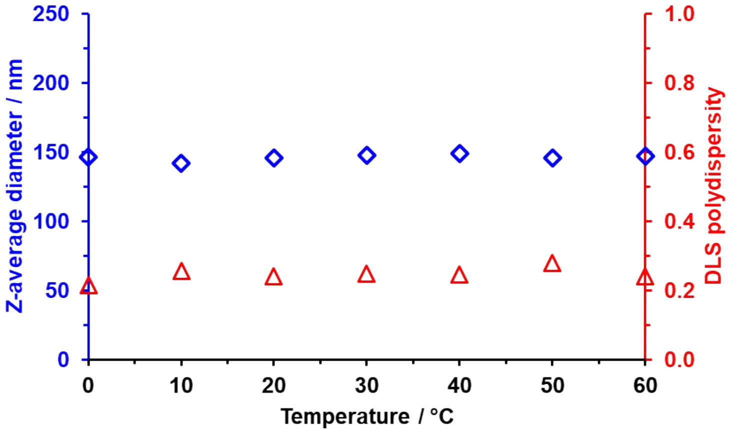

Figure S9. Variation in the sphere-equivalent hydrodynamic diameter (blue diamonds) and DLS polydispersity (red triangles) obtained for GA-crosslinked PEG_{113}-$PHBA_{300}$ worms prepared at 20 °C. The minimal change in each parameter confirms effective covalent stabilization. In contrast, the linear PEG113-PHBA300 nano-objects undergo inter-conversion between spheres, worms and vesicles over the same temperature change, as indicated in Fig. 7.

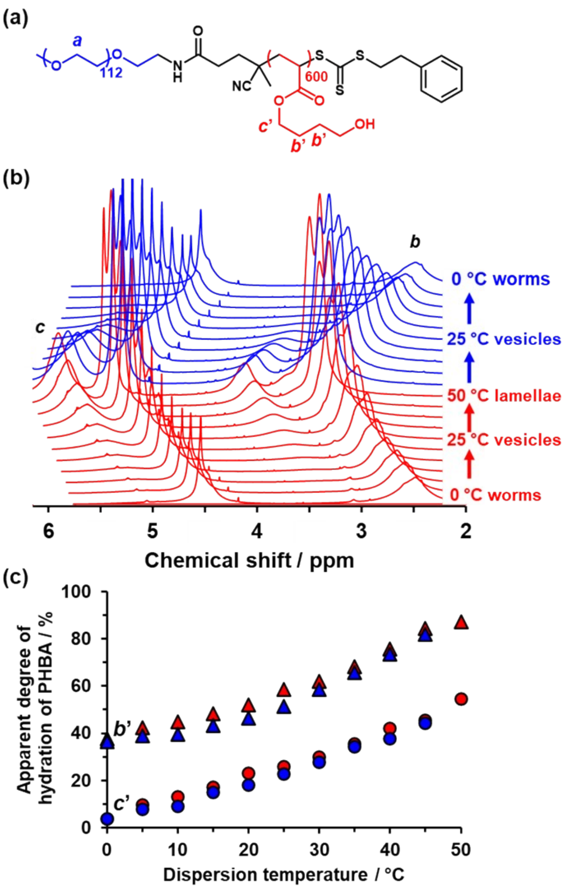

Figure S10. Variable temperature ^{1}H NMR studies of thermoresponsive linear PEG_{113}-$PHBA_{600}$ diblock copolymers with (a) assignment of the corresponding proton signals. (b) Normalized [relative to an external standard (pyridine)] partial ^{1}H NMR spectra recorded on heating from 0 °C to 50 °C. (c) Relative degrees of hydration calculated for the hydrophobic $PHBA_{600}$ block as a function of temperature when using proton signals *b*' and *c*'.

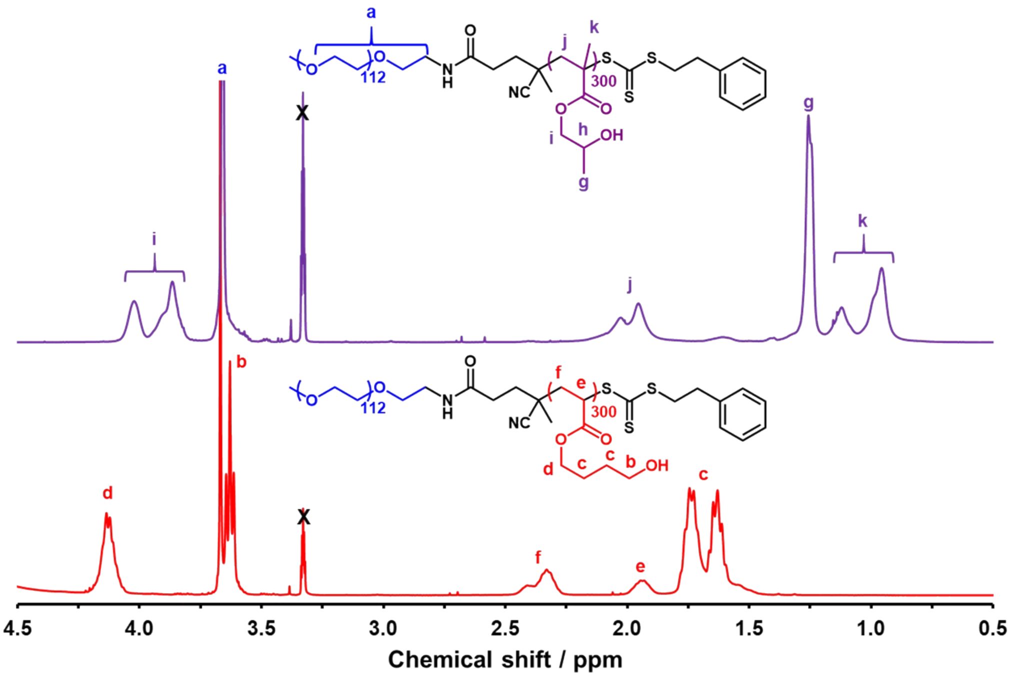

Figure S11. Assigned ^{1}H NMR spectra of PEG_{113}-$PHPMA_{300}$ (purple) and PEG_{113}-$PHBA_{300}$ (red) diblock copolymer chains in CD_3OD (marked with an **X** in the two spectra).

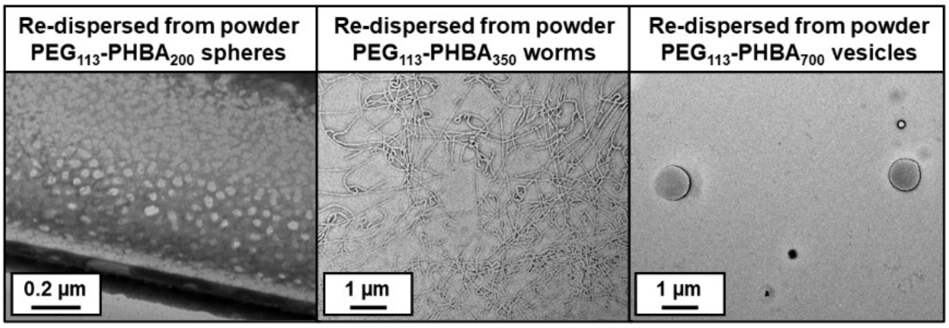

	Before	After
**Diblock copolymer composition**	**Diameter / nm (PDI)**	**Diameter / nm (PDI)**
**PEG_{113}-$PHBA_{200}$**	**44 (0.02)**	**43 (0.06)**
**PEG_{113}-$PHBA_{350}$**	**157 (0.28)**	**122 (0.34)**
**PEG_{113}-$PHBA_{700}$**	**588 (0.31)**	**680 (0.48)**

Figure S12. Representative TEM images obtained for 0.05% w/w aqueous dispersions of reconstituted (a) PEG_{113}–$PHBA_{200}$ spheres, (b) PEG_{113}–$PHBA_{350}$ worms, and (c) PEG_{113}–$PHBA_{700}$ vesicles. In each case, glutaraldehyde crosslinking (after reconstitution) was conducted for 16 h using 5.0% w/w aqueous dispersions of these nano-objects at GA/HBA = 0.66, pH 7 and 20 °C. In principle, the relatively high (partial) degree of hydration of PHBA plus its much lower T_g compared to PHPMA should facilitate direct reconstitution of diblock copolymer nano-objects from freeze-dried PEG_{113}-$PHBA_x$ powders. Indeed, 15% aqueous dispersions of PEG_{113}–$PHBA_{200}$ spheres, PEG_{113}–$PHBA_{350}$ worms and PEG_{113}–$PHBA_{700}$ vesicles can each be reconstituted via direct dissolution at pH 7 simply by stirring for 2 h at 20°C.

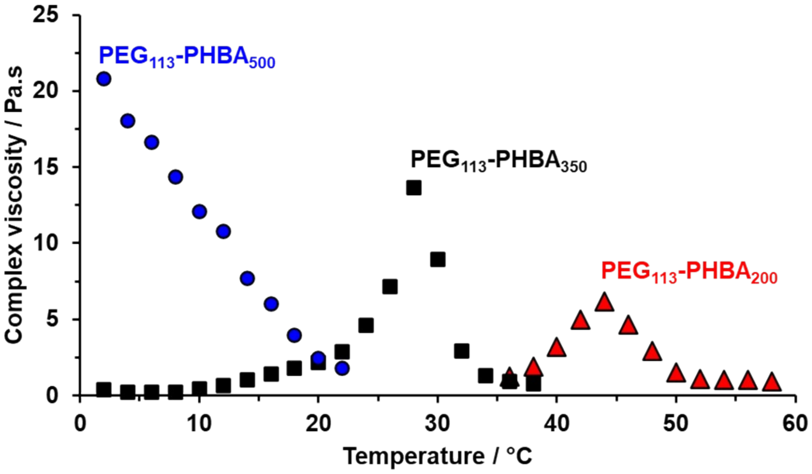

Figure S13. Temperature-dependent complex viscosity data obtained for 15% w/w aqueous dispersions of (a) PEG_{113}-$PHBA_{500}$ (blue circles), (b) PEG_{113}-$PHBA_{350}$ (black squares), and (c) PEG_{113}-$PHBA_{200}$ (red triangles) at an applied strain of 1.0% and an angular frequency of 1.0 rad s^{-1}. Each dispersion was equilibrated at 2 °C for 30 min prior to heating.

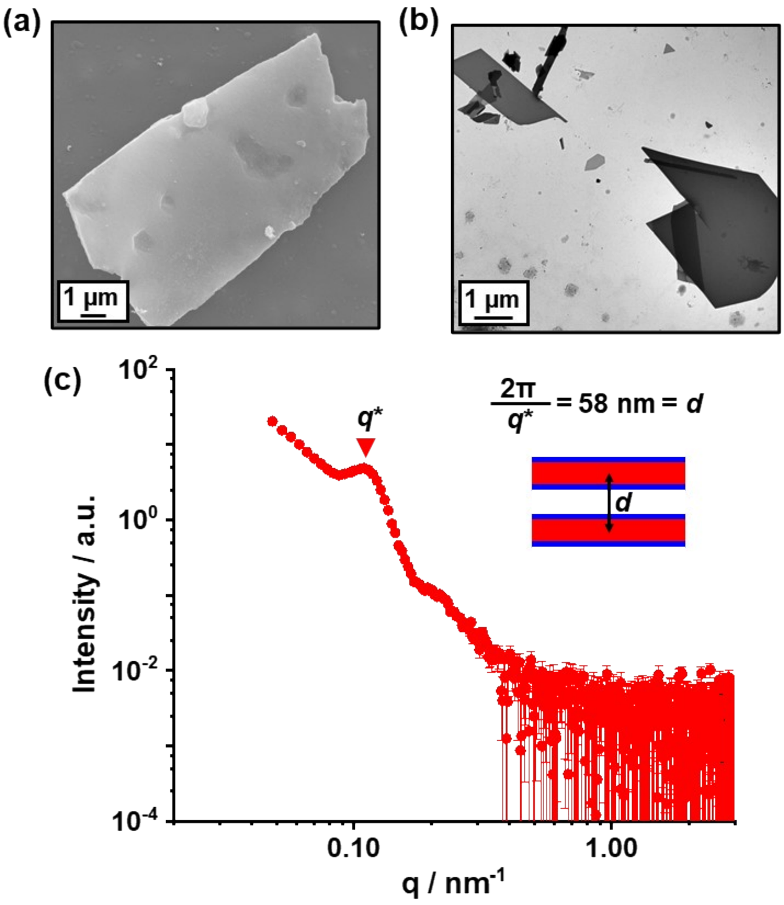

Figure S14. Representative (a) SEM and (b) TEM images of GA-crosslinked PEG_{113}-$PHBA_{500}$ diblock copolymer lamellae prepared at 50°C. Both imaging techniques provided strong evidence for the formation of relatively large (> 1 µm) lamellar sheets. (c) SAXS patterns recorded for a 1.0% w/w aqueous dispersion of linear PEG_{113}–$PHBA_{500}$ diblock copolymer lamellae at 50°C. The mean inter-sheet stacking distance was 115 nm was calculated from the diffraction peak labeled *q** using the equation shown in the inset.[26]

References

1 M. Save, J. V. M. Weaver, S. P. Armes and P. McKenna, *Macromolecules*, 2002, **35**, 1152–1159.

2 N. J. W. Penfold, J. R. Whatley and S. P. Armes, *Macromolecules*, 2019, **52**, 1653–1662.

3 M. Basham, J. Filik, M. T. Wharmby, P. C. Y. Chang, B. El Kassaby, M. Gerring, J. Aishima, K. Levik, B. C. A. Pulford, I. Sikharulidze, D. Sneddon, M. Webber, S. S. Dhesi, F. Maccherozzi, O. Svensson, S. Brockhauser, G. Náray and A. W. Ashton, *J. Synchrotron Radiat.*, 2015, **22**, 853–858.

4 J. S. Pedersen, *J. Appl. Crystallogr.*, 2000, **33**, 637–640.

5 J. S. Pedersen and M. C. Gerstenberg, *Colloids Surfaces A Physicochem. Eng. Asp.*, 2003, **213**, 175–187.

6 J. S. Pedersen, C. Svaneborg, K. Almdal, I. W. Hamley and R. N. Young, *Macromolecules*, 2003, **36**, 416–433.

7 D. J. Kinning and E. L. Thomas, *Macromolecules*, 1984, **17**, 1712–1718.

8 J. S. Pedersen and P. Schurtenberger, *Macromolecules*, 1996, **29**, 7602–7612.

9 Y. Takahashi and H. Tadokoro, *Macromolecules*, 1973, **6**, 672–675.

10 L. J. Fetters, D. J. Lohse, D. Richter, T. A. Witten and A. Zirkel, *Macromolecules*, 1994, 27, 4639–4647.

11 J. Ilavsky and P. R. Jemian, *J. Appl. Crystallogr.*, 2009, **42**, 347–353.

12 N. M. Ahmad, F. Heatley and P. A. Lovell, *Macromolecules*, 1998, **31**, 2822–2827.

13 F. Heatley, P. A. Lovell and T. Yamashita, *Macromolecules*, 2001, **34**, 7636–7641.

14 N. M. Ahmad, B. Charleux, C. Farcet, C. J. Ferguson, S. G. Gaynor, B. S. Hawkett, F. Heatley, B. Klumperman, D. Konkolewicz, P. A. Lovell, K. Matyjaszewski and R. Venkatesh, *Macromol. Rapid Commun.*, 2009, **30**, 2002–2021.

15 K. Shameli, M. Bin Ahmad, S. D. Jazayeri, S. Sedaghat, P. Shabanzadeh, H. Jahangirian, M. Mahdavi and Y. Abdollahi, *Int. J. Mol. Sci.*, 2012, **13**, 6639–6650.

16 Y. Huang, H. Ma, S. Wang, M. Shen, R. Guo, X. Cao, M. Zhu and X. Shi, *ACS Appl. Mater. Interfaces*, 2012, **4**, 3054–3061.

17 H. Zhu, M. Du, M. Zhang, P. Wang, S. Bao, Y. Fu and J. Yao, *Sensors Actuators B Chem.*, 2013, **185**, 608–619.

18 A. G. Destaye, C.-K. Lin and C.-K. Lee, *ACS Appl. Mater. Interfaces*, 2013, **5**, 4745–4752.

19 H. Zhu, M. Zhang, S. Cai, Y. Cai, P. Wang, S. Bao, M. Zou and M. Du, *RSC Adv.*, 2014, **4**, 794–804.

20 R. Rudra, V. Kumar and P. P. Kundu, *RSC Adv.*, 2015, **5**, 83436–83447.

21 H. S. Mansur, C. M. Sadahira, A. N. Souza and A. A. P. Mansur, *Mater. Sci. Eng. C*, 2008, **28**, 539–548.

22 A. Czajka and S. P. Armes, *Chem. Sci.*, 2020, **11**, 11443–11454.

23 G. Battaglia and A. J. Ryan, *J. Am. Chem. Soc.*, 2005, **127**, 8757–8764.

24 J. R. Lovett, L. P. D. Ratcliffe, N. J. Warren, S. P. Armes, M. J. Smallridge, R. B. Cracknell and B. R. Saunders, *Macromolecules*, 2016, **49**, 2928–2941.

25 N. J. Warren, M. J. Derry, O. O. Mykhaylyk, J. R. Lovett, L. P. D. D. Ratcliffe, V. Ladmiral, A. Blanazs, L. A. Fielding and S. P. Armes, *Macromolecules*, 2018, **51**, 8357–8371.

26 M. Li, Y. Liu, H. Nie, R. Bansil and M. Steinhart, *Macromolecules*, 2007, **40**, 9491–9502.
